# NanoString Technology for Human Papillomavirus Typing

**DOI:** 10.3390/v13020188

**Published:** 2021-01-27

**Authors:** Mangalathu S. Rajeevan, Sonya Patel, Tengguo Li, Elizabeth R. Unger

**Affiliations:** Centers for Disease Control and Prevention, Division of High-Consequence Pathogens & Pathology, 1600 Clifton Road, Atlanta, GA 30329, USA; sqp1@cdc.gov (S.P.); uyy7@cdc.gov (T.L.); eru0@cdc.gov (E.R.U.)

**Keywords:** NanoString, HPV detection, Linear Array, TypeSeq, PCR cycles

## Abstract

High-throughput HPV typing assays with increased automation, faster turnaround and type-specific digital readout would facilitate studies monitoring the impact of HPV vaccination. We evaluated the NanoString nCounter^®^ platform for detection and digital readout of 48 HPV types in a single reaction. NanoString (NS) used proprietary software to design CodeSets: type-specific probe pairs targeting 48 HPV types and the globin gene. We tested residual DNA extracts from epidemiologic specimens and defined samples (HPV plasmids at 10 to 10^4^ copies/reaction) directly (No-PCR) as well as after L1 consensus PCR of 45 (PCR-45) or 15 cycles (PCR-15). Assay and interpretation followed NS recommendations. We evaluated analytic performance by comparing NanoString results for types included in prior assays: Roche Linear Array (LA) or HPV TypeSeq assay. No-PCR results on 40 samples showed good type-specific agreement with LA (k = 0.621) but sensitivity was 65% with lower limit of detection (LOD) at 10^4^ plasmid copies. PCR-45 results showed almost perfect type-specific agreement with LA (k = 0.862), 82% sensitivity and LOD at 10 copies. PCR-15 results on 75 samples showed substantial type-specific agreement with LA (k = 0.796, 92% sensitivity) and TypeSeq (k = 0.777, 87% sensitivity), and LOD at 10 copies of plasmids. This proof-of-principle study demonstrates the efficacy of the NS platform with HPV CodeSet for type-specific detection using a low number of PCR cycles (PCR-15). Studies are in progress to evaluate assay reproducibility and analytic validation with a larger number of samples.

## 1. Introduction

The introduction of prophylactic HPV vaccines has the potential to reduce the global burden of HPV-associated cervical, anogenital and a subset of oropharyngeal cancers. Reduction in population-based, type-specific prevalence of vaccine-targeted types is an early biologic indicator of the impact of HPV vaccination. Large surveillance studies have documented robust trends in the expected decrease in vaccine targeted types in more than 14 countries with HPV vaccination programs [1], including the US [2]. In addition, these studies also monitor the potential risk of HPV type-replacement [3,4] on the basis of evolution of higher virulence in response to vaccine immunity [5], type-competition and diagnostic artifacts [6,7] or co-infection and phylogenetic relatedness of non-vaccine types [8]. Assays approved by the US Food and Drug Administration (FDA) for clinical use cover only 13–14 high-risk (HR) types and many do not provide type-specific results [9]. Thus, HPV vaccine surveillance studies frequently rely on Research Use Only tests. The CDC’s HPV surveillance studies have relied on the now discontinued Roche Linear Array HPV Assay (LA; Roche Diagnostics, Indianapolis, IN) [10,11,12]. An ideal replacement assay would have multiplexing capability to detect and individually identify at least the 37 alpha types in LA, with comparable type-specific sensitivity and internal controls. HPV surveillance assays should also be high throughput, have increased automation, faster turnaround time and digital read out for seamless accurate data transfer. The recently reported TypeSeq assay has many of these characteristics and results are comparable with LA [13]. It is a cost-effective HPV L1 amplicon-based Next Generation Sequencing (NGS) assay targeting 51 alpha types with high throughput. However, it requires multiple rounds of PCR using hundreds of primers, sample pooling and customized bioinformatics pipeline for automated calling of types. In this proof-of principle study, we evaluated the analytic performance of NanoString (NS) Technology’s (Seattle, WA) nCounter^®^ platform [14] for digital readout of 48 HPV types in a single reaction without sample pooling. Results of HPV typing by NS technology, with or without L1 PCR, were evaluated for concordance with results from LA and TypeSeq assays. This study demonstrates the potential of using the NS platform for highly multiplexed, high-throughput detection and typing of HPV with a low number of PCR cycles.

## 2. Materials and Methods

### 2.1. Overview of Study 

**Figure 1:**
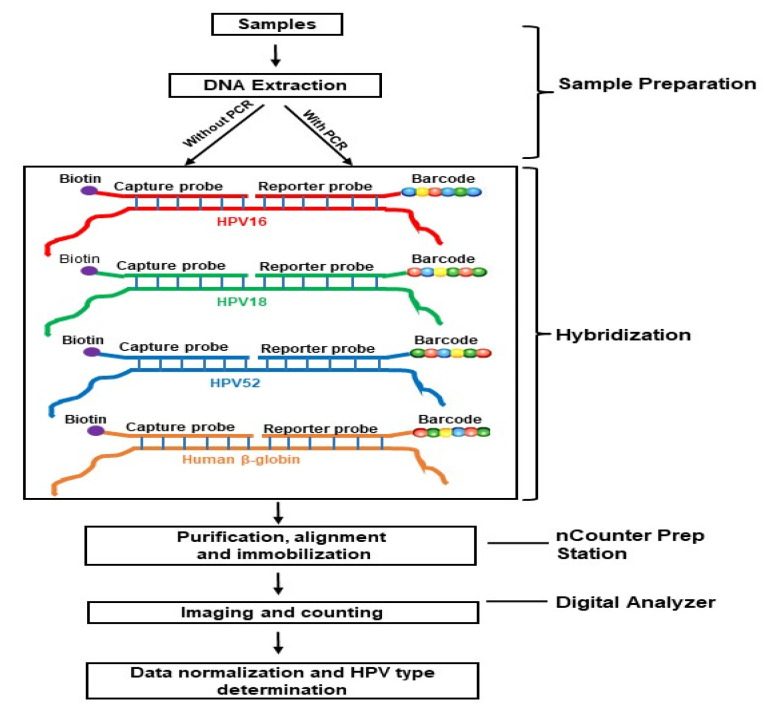
A schematic illustration of the conceptual framework using a sample with multiple infection of HPV types 16, 18 and 52 to evaluate NanoString technology for HPV typing. A CodeSet for an HPV type consists of sequence-specific capture and reporter probes, each 35–50 bases long. The CodeSets are hybridized to DNA extract, either directly or to amplified product. Following hybridization, the tripartide complex is purified, aligned and immobilized in a sample cartridge using the automated nCounter Prep Station. Cartridges are then transferred to the Digital Analyzer for imaging and digital counting of molecular barcodes carried in the reporter probes. HPV type determination is based on normalized count of barcodes that pass a cut-off.

Figure 1 illustrates the conceptual framework of the study evaluating the use of NS Technology to detect and type HPV. We evaluated the NS [14] assay using DNA extracts without any amplification (No-PCR) or after amplifying HPV targets with 45 (PCR-45) or 15 cycles of PCR (PCR-15). The assay follows the NS strategy, using hybridization to CodeSets that consist of pairs of capture and reporter probes, each 35–50 bases long. The reporter probe carries a unique molecular barcode for detection (six positions and four colors allowing 4 × 10^6^ = 4096 unique barcodes). The capture probe carries biotin for immobilization of purified tripartide complexes to the sample cartridge using the automated nCounter Prep station. Sample cartridges are then placed into the Digital Analyzer for imaging and digital counting of barcodes for HPV type determination. Detection requires hybridization of both the capture and reporter probes, providing increased specificity.

### 2.2. CodeSets 

Two different versions of CodeSets were prepared, each targeting 48 HPV types. The types included all 37 detected by LA [15] (HPV6, 11, 16, 18, 26, 31, 33, 35, 39, 40, 42, 45, 51, 52, 53, 54, 55, 56, 58, 59, 61, 62, 64, 66, 67, 68b, 69, 70, 71, 72, 73, 81, 82, 82 subtype IS39, 83, 84, 89), and 11 additional types (HPV13, 30, 34, 43, 44, 68a, 74, 87, 90, 91, 114) detectable by other methods [16,17]. Among the 48 HPV types, 14 are considered HR types (**HPV16, 18, 31, 33**, 35, 39, **45**, 51, **52**, 56, **58**, 59, 66, 68b—types in bold represent the seven nonvalent HR vaccine types) [18], and are included in clinical HPV tests that have been FDA-approved in the US. Version 1, used for direct hybridization of DNA extracts (No-PCR), included a total of 97 CodeSets targeting both L1 (Supplementary Table S1) and E6 (Supplementary Table S2) regions, along with two CodeSets for *HBB* (GenBank ID: GU324922.1). Due to sequence constraints, an E6 CodeSet for HPV64 could not be prepared, otherwise each type was targeted in both L1 and E6 regions. Version 2, used for amplified products (PCR-15 or PCR-45), included a total of 49 CodeSets, 48 targeting the 450 bp region amplified by L1 consensus PCR [19] and one targeting the amplified 268 bp *HBB* sequence [20] (Supplementary Table S3). CodeSets were designed by NS using their proprietary software and HPV reference sequences from the PapillomaVirus Episteme database (PaVE; https://pave.niaid.nih.gov/#home) for all the selected types, except that GenBank IDs U31791.1, AJ812226.1, FR751039.1 and AF29396.1 were used for HPV55, 64, 68b and 82 subtype IS39, respectively. Probes were designed within more conserved regions among variants within each HPV type, and none of the CodeSets showed potential for cross-hybridization and secondary structure or with melting temperature (Tm) and GC content out of range. All CodeSets were custom-made following standard CodeSet chemistry by NS. Briefly, Integrated DNA Technologies (IDT, Coralville, IA, USA) synthesized DNA oligos (target-specific region plus 9 nucleotide linkers for each capture and reporter probe) with standard desalting purification. These oligos were then shipped to NS to perform ligation reactions in-house and assemble the various probes into a single CodeSet (contact authors for custom ID’s of these CodeSets) specific to an HPV type (1 CodeSet = 1 HPV type).

The assay also includes manufacturer-designed CodeSets for internal positive and negative controls. These were designed against 14 External RNA Controls Consortium (ERCC) transcript sequences (6 positive and 8 negative hybridization controls). For each ERCC positive control, in vitro transcribed RNAs were premixed, each one at 128, 32, 8, 2, 0.5 and 0.125 fM concentrations. ERCC target transcripts were absent in negative controls.

### 2.3. Samples

Defined samples of known HPV copy number (HPV plasmid pools prepared in placental DNA at 100 ng as carrier, DNA extracts of HPV16-positive SiHa and HPV18-positive HeLa cell lines) and HPV-negative controls (placental DNA and water) were included to estimate assay sensitivity. In addition, we used residual anonymized DNA extracts from epidemiologic studies of HPV with previous LA typing results. These samples were from a variety of anogenital sites (cervical, vaginal and anal), and included extracts from cells in diverse collection media (SurePath preservative fluid (Becton Dickinson Co., Franklin Lakes, NJ, USA), Specimen Transport Medium (STM, Qiagen, Germantown, MD, USA, foam tipped dry swab (Puritan, Guilford, ME, USA) as well as formalin-fixed paraffin-embedded (FFPE) tissue. The extraction methods were matched to sample type and included manual (QiaAmp and DNeasy, Qiagen, Germantown, MD, USA) and automated methods (Chemagic MSM1 extractor (Perkin Elmer Waltham, MA, USA, MagNA Pure LC System (Roche Applied Science, Indianapolis, IN, USA)). Extracts were stored for at least 5–10 years at –80 °C prior to NS testing. 

The 48 samples tested directly (No-PCR) included eight defined samples (HPV plasmid pools including 5 types (HPV11, 16, 31, 45, 52) in copy numbers from 10 to 10^4^ per reaction, SiHa (100 and 10 ng/reaction), placental DNA and water), 25 residual DNA extracts from cervix (15 FFPE and 5 each SurePath and STM), 5 cervico-vaginal (self-collected dry swabs) and 10 anal (STM) (Supplementary Table S4). The 48 samples for PCR-45 included the identical distribution of sample types, but different extracts were used. Based on results from these two trials of No-PCR and high-cycle PCR conditions, we selected conditions with low-cycle PCR for further evaluation as No-PCR appeared to have limited sensitivity and PCR-45 required >200,000-fold dilution for analysis. We examined 10, 15 and 20 cycles and selected 15 cycles based on detection of HPV plasmid DNA at 10 copies. We further evaluated PCR-15 with 75 samples including 7 defined samples (HPV plasmid pools including 7 types (HPV6, 16, 18, 31, 33, 45, 52) in copy numbers/reaction from 10 to 50, SiHa and HeLa (each 10 ng/reaction), placental DNA and water), 30 residual DNA extracts from cervix (20 FFPE and 10 SurePath), 20 cervico-vaginal (self-collected dry swabs) and 18 anal (STM) (Supplementary Table S5). 

### 2.4. Sample Preparation

For direct hybridization (No-PCR), 50 µL of DNA extract was sheared to 200–300 bp using sonication (Covaris M220 SonoLab 7.1.4) at NS Laboratory, then precipitated with ethanol, resuspended in 10 µL Tris (pH 8.0), denatured for 5 min at 95 °C, and snap cooled on ice. For NS using products of amplification, PGMY09/11 L1 consensus PCR amplicons were prepared following published protocols [19,21], with the addition of primer RSMY09-L to improve the detection of HPV type 68a [22]. Briefly, the 50 µL reaction included 5 µL of DNA extract, 1x PCR buffer II, 0.2 mM dNTP, 3 nM MgCl_2_, 1.25U of AmpliTaq Gold, 80 nM each of PGMY9, PGMY11, HMB01 and RSMY09-L primers and 20 nM each of beta-globin (*HBB*) primers GH20 and PCO4 [20] (Supplementary Table S6). Amplification started with a pre-heat at 95 °C for 9 min, followed by 15 or 45 amplification cycles (95 °C for 30 s, 55 °C for 1 min and 30 s, and 72 °C for two minutes), ending with a final extension for five minutes at 72 °C. PCR products were stored at −20 °C until testing at the NS Laboratory, where the DNA concentration of amplicons from PCR-45 was determined with Qubit fluorometer, and amplicons were diluted to final concentration of 450 fM (>200,000-fold dilution). The amplicons from PCR-15 were used directly. PCR products (without any clean-up or further fragmentation) were denatured at 95 °C for 5 min and snap cooled on ice.

### 2.5. Hybridization and Imaging

All hybridizations were done in a total volume of 15 µL (5 µL of denatured DNA added to master mix of 2 µL capture probe and 8 µL reporter probes suspended in hybridization buffer). Samples were hybridized at 65 °C for 16 h. Following hybridization, the tripartide complexes were purified, immobilized and imaged using the nCounter MAX System (includes both nCounter Prep Station and Digital Analyzer), to generate digital counts of barcodes corresponding to each target in the multiplexed reaction. Labeled barcodes obtained from unamplified extracts (No-PCR) were counted at 555 images or field of view (FOV) and PCR-15 and PCR-45 amplicons were counted at 280 FOV. The nCounter Digital Analyzer system scans each lane into a few hundred imaging sections called FOVs. The FOV number can be selected from one of the given options (25, 100, 280 and 550), with higher numbers giving better resolution but requiring more time. The barcode counts for each sample were recorded in Reporter Code Count (RCC) files that are imported into nSolver analysis software (provided with CodeSet by NS) for quality control evaluation. The quality control (QC) metrics included comparison of FOV attempted and successful counts (should be ≥75%; lower values indicate inability to focus), binding density (acceptable range 0.05–2.25) and scaling factors (acceptable range 0.03–3.0).

### 2.6. HPV TypeSeq Assay 

Results of the HPV TypeSeq assay, a recently developed amplicon-based NGS assay targeting 51 HPV types [13], were available for 44 of the 75 PCR-15 samples (Supplementary Table S5). The assay was conducted as described by Wagner et al. [13]. The library products were sequenced using the MiSeq system with 150 cycle v3 chemistry (160 × 13 bp) (Illumina, San Diego, CA, USA) with sequence analysis and HPV genotyping calls using a custom-built bioinformatics pipeline [13].

### 2.7. Data Analysis and Statistics

HPV type-specific hybridization signals were normalized against the ERCC positive and negative controls, and background assessed as per NS recommendations. Briefly, this involved first calculating a sample-specific scaling factor by dividing the average geomean of ERCC positive controls in all samples by geomean of ERCC positive controls in each specific sample. All negative controls and target-specific signal values were then normalized by multiplying these values with their sample-specific scaling factor. A positive target signal by the NS assay was defined as specimen normalized counts greater than the calculated sample-specific background (average of all ERCC negative normalized counts in a sample + 2 standard deviation). 

NS type-specific or sample concordance was evaluated by comparing results with LA or TypeSeq in 2 × 2 tables, restricting analysis to types included in both assays (No-PCR vs. LA or PCR-45 vs. LA or PCR-15 vs. LA or PCR-15 vs. TypeSeq). Type-specific concordance was reported for all types evaluated (defined as overall type-specific concordance) or by individual HPV type for all samples. For sample level comparisons, HPV-positive was defined as detection of one or more HPV types, and agreement rate was calculated as the sum of the number of samples positive for both tests and the number of negative by both tests, divided by the total number of specimens tested. Concordance at the sample was also defined based on the degree of matching between types detected in each assay, with full concordance indicating both assays agree for all HPV types, partial concordance indicating assay agreement on detection of at least one HPV type but disagreement for others, and full discordance indicating lack of assay agreement on any type. The agreement rate, kappa coefficient (k) with 95% confidence interval (95% Cl) and McNemar’s *p*-value were also calculated to measure concordance between tests. The kappa values were interpreted as poor (k < 0.20), fair (k = 0.21–0.40), moderate (k = 0.41–0.6), substantial (k = 0.61–0.80) and almost perfect (k = 0.81–1.00) agreement [23]. Following a previously defined method for HPV typing [24,25], the proportion of positive agreement (*Ppos*) for each type was calculated as (twice the number of agreed positives)/(total number of specimens + number of agreed positives − number of agreed negatives). GraphPad (https://www.graphpad.com/quickcalcs/) was used to perform statistical analysis. Two-sample proportion Z-test (https://www.statskingdom.com/121proportion_normal2.html) was used to test for significant differences between kappa coefficients or between agreement rates calculated from 2 × 2 tables. 

## 3. Results

### 3.1. Results of Direct Testing of DNA Extracts (No-PCR)

The NS QC metrics for all 48 samples used as direct DNA extracts (No-PCR) with CodeSet version 1 were within an acceptable range: FOV (529–555), binding density (0.1–2.08) and scaling factor (0.76–1.48). Placental DNA and the water control were negative with all HPV-specific CodeSets. Excluding the water control, 83% of samples (39/47) were positive for *HBB* (human DNA control). Concordance between E6 and L1 results in Version 1 CodeSet ranged from 55.32% to 100% (mean 94.72%), and all samples had full concordance (52%, 25/48) or partial concordance. Given the substantial agreement between E6 and L1 results (94.73%; k = 0.734; 95% CI = 0.688–0.779; McNemar *p*-value = 0.463) (Supplementary Table S7), L1 results were used in further analysis of No-PCR results. 

SiHa cell line DNA (10–100 ng) and plasmid pools for HPV11, 16, 31, 45, 52 (10–10,000 copies) were all positive for L1 but only at the highest input level tested. For the 40 epidemiologic samples, the type-specific concordance was 90% (Table 1) (k = 0.621, considered substantial; 95% CI = 0.562–0.679; McNemar *p*-value = 0.342). The type-specific sensitivity of No-PCR was 65% that of LA and the proportion of positive agreement between the No-PCR and LA results was 67%. The proportion of positive agreement and sensitivity slightly improved when concordance was restricted to 14 HR HPV types (73% positive agreement, 72% sensitivity) or HPV16/18 only (84% positive agreement, 81% sensitivity) (Table 1). Among the 40 epidemiological samples, LA and No-PCR results at the level of overall HPV detection agreed in 90% (36/40; 31 HPV-positive, 5 HPV-negative; k = 0.660, 95% CI = 0.363–0.957; McNemar *p*-value = 0.125). Among the concordant HPV-positive samples, all showed either full (13) or partial (18) type-specific agreement. While LA detected HPV in slightly more samples than No-PCR (35 versus 31, respectively), the mean number of HPV types detected per HPV-positive sample was similar between the assays (6 types/sample). 

### 3.2. Results of PCR-45 Test

The NS QC metrics for all 48 samples tested after 45 cycles of PCR were within an acceptable range: FOV (256–280), binding density (0.13–0.35) and scaling factor (0.793–1.453). Placental DNA and the water control were negative with all L1 CodeSets. Excluding the water control, 100% of samples (47/47) were positive for *HBB* (human DNA control). SiHa (10–100 ng) and plasmid pools for HPV16, 31, 45 and 52 (10–10,000 copies) were all positive with CodeSet version 2 (L1 CodeSet). 

For the 40 epidemiologic samples, the type-specific concordance with LA was 96.2% (Table 2) (k = 0.86, (95% CI = 0.828–0.898) considered almost perfect agreement; McNemar *p*-value < 0.000001), the type-specific sensitivity was 82% and the proportion of positive agreement was 89%. The proportion of positive agreement and sensitivity increased evaluating concordance restricted to 14 HR types (93% positive agreement and 87% sensitivity) or to HPV 16/18 only (100% positive agreement and 100% sensitivity) (Table 2). At the sample level, LA and PCR-45 results for overall HPV detection showed complete agreement (36 HPV-positive, 4 HPV-negative; k = 1.00 (95% CI = 1.00–1.00)) and all positive samples showed full (16) or partial (20) type concordance. Among the HPV-positive samples, the mean number of types per sample was slightly higher for LA compared with PCR-45 (7.20 versus 6.13, respectively). 

### 3.3. Results of PCR-15 Test

The NS QC metrics for all 75 samples tested after 15 cycles of PCR were within an acceptable range: FOV (279–280), binding density (0.17–1.65) and scaling factor (0.65–2.31). Excluding the water control, 97% of samples (72/74) were positive for *HBB* (human DNA control). SiHa (10 ng), HeLa (10 ng) and plasmid pools for HPV6, 11, 16, 18, 31, 33, 45 and 52 (10–50 copies) were all positive with L1 CodeSets version 2.

**Figure 2:**
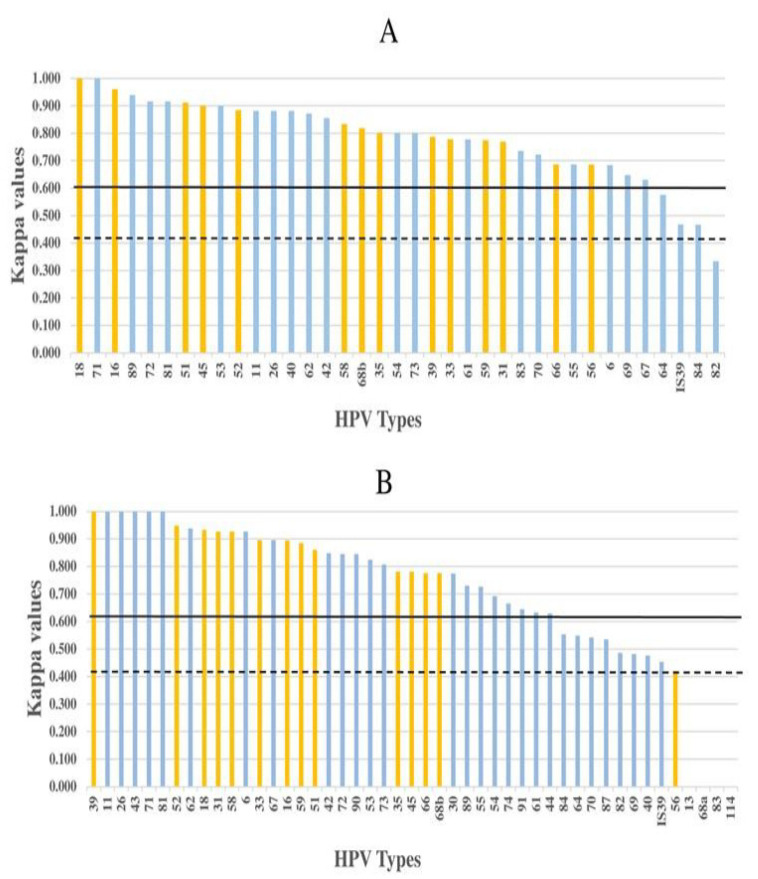
Individual HPV type-specific agreement as indicated by kappa values between PCR-15 and LA for 37 types (**A**), and between PCR-15 and TypeSeq for 47 types (**B**). HPV types are shown on the X-axis (yellow bars: HR types and others: LR types) and corresponding kappa values on the Y-axis. HPV types with kappa values crossing the black line represent >substantial agreement (k ≥ 0.61) between tests, whereas HPV types that fall between the black line and dashed black line represent moderate (k = 0.41–0.6) agreement between tests.

For the 75 samples (7 defined and 68 epidemiological), the type-specific concordance with LA was 95.7% (Table 3, all types) (k = 0.796 (95% CI = 0.761–0.832), considered substantial; McNemar *p*-value < 0.000001) with 92% and 96.2% type-specific sensitivity and specificity respectively, and 82% proportion of positive agreement. The proportion of positive agreement and sensitivity increased when concordance was restricted to 14 HR types (87% positive agreement and 96% sensitivity and specificity), to the 7 nonvalent HR vaccine types (89% positive agreement, 98% sensitivity and 97% specificity) or to HPV16/18 (98% positive agreement, 100% sensitivity and 99% specificity). Individual type-specific agreement ranged from 86% to 100% (k-values 0.334–1.00) and 92% (34/37) of the LA types showed agreement > 90%. All HR types showed >90% agreement (k-values 0.686–1.00, considered substantial to perfect; Table 3). All types except types 64, 82, 82 subtype IS39 and 84 showed substantial type-specific concordance (k-values ≥ 0.61) (Figure 2A). Types 64, 82 subtype IS39 and 84 showed moderate agreement (k-values 0.41–0.576), whereas agreement between tests for the type 82 was only fair (k = 0.334). 

At the sample level, LA and PCR-15 results for overall HPV detection showed near complete agreement (74/75 (99%), 69 HPV-positive, 5 HPV-negative; k = 0.902 (95% CI = 0.742–1.00)), 98.5% sensitivity and 100% specificity. All but one of the HPV concordant positive samples showed full (48) or partial (20) type agreement. Among the HPV-positive samples, the mean number of types per sample was slightly lower for LA compared with PCR-15 (4.2 versus 5.3, respectively). 

### 3.4. Concordance between PCR-15 and HPV TypeSeq

For the subset of 44 samples with TypeSeq results, the type-specific concordance with PCR-15 for all 47 types was 95% (Table 4) (k = 0.777, considered substantial; 95% CI = 0.736–0.888; McNemar *p*-value < 0.000178). The type-specific sensitivity and specificity of PCR-15 were 89% and 96% to that of TypeSeq and the proportion of positive agreement between PCR-15 and TypeSeq was 81%. The proportion of positive agreement and sensitivity increased when concordance was restricted to 14 HR types (89% positive agreement, 92% sensitivity and 96% specificity) or to the 7 nonvalent HR vaccine types (92% positive agreement, 97% sensitivity and 97% specificity) or to HPV 16/18 only (93.6% positive agreement, 100% sensitivity and 95.5% specificity). Individual type-specific agreement ranged from 82% to 100%, with 91% (43/47) of the types showing agreement > 90% (Table 4). Among the 47 types, k-values for 34 types (72%) showed substantial to perfect agreement (k ≥ 0.61–1.00), 9 types showed moderate agreement (k-value 0.421–0.553) and 4 types (types 13, 68a, 83 and 114) showed poor agreement (k < 0.20) between tests (Figure 2B). All HR types except HPV 56 showed >90% agreement (k-values 0.776–0.949, substantial to perfect agreement). HPV 56 showed 86.4% agreement (k = 0.421, moderate agreement) (Table 4 and Figure 2B). At the sample level, TypeSeq and PCR-15 results for overall HPV detection showed complete agreement (41 HPV-positive, 3 HPV-negative, k = 1.00 (95% CI = 1.00–1.00)) and both sensitivity and specificity at 100%. All the HPV concordant positive samples showed full (24) or partial (17) type agreement. Among the HPV-positive samples, the mean number of types per sample was slightly lower for TypeSeq compared with NS PCR-15 (6.2 versus 7.1, respectively). 

### 3.5. Z-Test Results

As evaluated by the kappa coefficients and agreement rates with LA, PCR-15 and PCR-45 did not show significant differences in performance by the two-sample Z-test (all *p*-values > 0.05; Supplementary Table S8). In addition, Z-test results indicated that PCR-15 type-specific concordance with LA was not significantly different from PCR-15 type-specific concordance with TypeSeq, as indicated by the lack of significant difference between kappa coefficients or between the agreement rates (Supplementary Table S8). Lack of significance in *p*-value is also supported by the overlap in 95% CI of kappa coefficients. 

## 4. Discussion

This is the first report of a novel approach for type-specific detection of HPV in a single reaction with almost perfect agreement to the 14 HR types (including the seven nonvalent HR vaccine types) using NS technology with no or minimal cycles of PCR. Both positive and negative controls behaved as expected in all formats of NS assay tested (No-PCR with CodeSet Version 1; PCR-15 and PCR-45 with CodeSet Version 2). There was substantial concordance between No-PCR and LA both at sample level detection and overall type-specific agreement (90% each). However, with No-PCR, HPV plasmid pools required at least 10^4^ copies, and SiHa at 100 ng/reaction for detection of HPV. This was reflected in the low sensitivity of No-PCR compared to LA (65% accounting for all 37 LA types). The sensitivity slightly improved when analysis was restricted to 14 HR types (72%) or HPV16/18 only (81%). While the NS platform was able to detect a number of HPV types in epidemiological samples with No-PCR, its sensitivity was not comparable to other methods that incorporate target or signal amplification [9,26]. This suggested that sensitivity of the NS platform could be improved with the addition of PCR. 

Given the agreement between the E6 and L1 CodeSets, we used L1 PCR with PGMY 9/11 primers generating the same 450 bp amplicon as LA. NS with 45 cycles of PCR (PCR-45) dramatically increased the sensitivity to detection of HPV plasmid pools at 10 copies/reaction and detection of *HBB* in all samples (including SiHa 10 ng/reaction). As shown in Table 2, type-specific agreement and sensitivity in comparison to LA also increased. In terms of agreement at sample level HPV detection, PCR-45 was 100% concordant with LA.

The NS assay with 15 cycles of PCR (PCR-15) was validated using 75 samples (68 epidemiological samples and 7 defined samples, details in Supplementary Table S5). Excluding the water control, 97% of samples (72/74) were positive for the human DNA endogenous control (*HBB*). Detection of human genomic DNA is important to verify sample adequacy. Overall type-specific concordance and sensitivity of PCR-15 compared to LA were 95.7% and 92% respectively, about a 27% increase in sensitivity from No-PCR to PCR-15. Like PCR-45, PCR-15 also resulted in almost perfect type-specific concordance (96.2–99.3%) and high sensitivity (96–100%) when the analysis was restricted to 14 HR HPV types (k = 0.848; 95% CI = 0.802–0.894) or to the seven nonvalent HR vaccine types (k = 0.89; 95% CI = 0.837–0.943) or to HPV16/18 only (k = 0.979; 95% CI = 0.939–1.00). Again, like PCR-45, PCR-15 was nearly 100% concordant with LA for detection of HPV at the sample level. TypeSeq results were available for 44 of the samples tested with PCR-15. There was substantial overall type-specific concordance (95%) and sensitivity (89%) for the 47 types in common between the two assays. Sensitivity for PCR-15 relative to TypeSeq ranged from 93% to 100% when the analysis was restricted to 14 HR types or HPV16/18, respectively. At the sample level, detection of HPV was fully concordant between PCR-15 and TypeSeq. The agreement between PCR-15 and TypeSeq is similar to the level of agreement reported between TypeSeq and LA when analysis was restricted to 14 HR types (k = 0.862; 95% CI = 0.811–0.912) or to the seven nonvalent HR types (k = 0.907; 95% CI = 0.850–0.963) or to HPV16/18 (k = 0.913; 95% CI = 0.817–1.00), respectively [13]. 

The two-sample Z-test indicated that there were no significant differences (all *p*-values > 0.05; Supplementary Table S8) in the type-specific agreement rates determined for PCR-45 vs. LA, PCR-15 vs. LA and PCR-15 vs. TypeSeq. Also, no significant difference in the kappa coefficient was indicated by the overlap in their 95% CI (Supplementary Table S8). Supported by the Z-test results, we conclude that performance of HPV CodeSets is highly specific and reproducible regardless of the differences in PCR cycle number and diversity of samples (different anatomical sites, varying collection media and fixatives, varying extraction methods). In addition, PCR-15 is the preferred protocol for further validation studies since the products from PCR-15 could be hybridized directly, eliminating errors during dilution, and fewer amplification cycles have the advantage of reducing amplification bias in type detection [26]. The mean numbers of HPV types detected per sample by PCR-15 (5.3), LA (5.8) and TypeSeq (6.3) were similar, further supporting the comparability of the PCR-15 assay.

PCR-15 showed moderate to almost perfect agreement with LA or TypeSeq for most individual types except for types 13, 68a, 83 and 114. HPV83 deserves further evaluation as PCR-15 showed substantial agreement (75% positive agreement) with LA but poor agreement with TypseSeq, similar to previously reported discordance (40% positive agreement) between LA and TypeSeq for this type [13]. PCR-15 detected HPV13 in 2 out of 44 samples in this study but TypeSeq did not detect HPV13 in the larger set of the SUCCEED study with 849 specimens [13]. Both PCR-15 and TypeSeq should detect types 68a and 114, but in this study, only PCR-15 detected these types (Table 4). Additional testing is required to refine assay performance, particularly for a few of these non-LA LR types as the differences could still be related to the small numbers of these types or due to assay differences. NS assays require hybridization of both capture and reporter probes for detection of specific targets (14). TypeSeq utilizes rhPCR primers [27] in Stage 1 PCR for type-specificity but rhPCR primers may be more sensitive to unknown nucleotide variants leading to type “drop-out” (13). Increased detection of HPV68a with PCR-15 may reflect the addition of RSMY09-L primer to the PGMY09/11 primer pool [28]. 

Currently, the importance of the non-LA LR types may be debatable but a few of these types (HPV30, 44, 68a, 87, 90, 114) were detected in at least 5–8 samples by PCR-15. TypeSeq also detected a few of these non-LA LR types (HPV30, 43, 74, 87, 90, 91, 114) in the SUCCEED study that tested 2804 specimens [29]. These results suggest that these non-LA LR types could be useful in the evaluation of HPV-negative cervical lesions [30] and in epidemiologic studies of HPV in geographical regions that may have uncommon types in circulation [31]. 

Compared to other common HPV assays with extended type-specific results for epidemiologic surveillance (LA, Novaplex or TypeSeq), PCR-15 has some additional attractive features. The assay has a simple automated workflow with limited hands-on time and provides direct read out of individual types from a single reaction. With only 15 PCR cycles, amplification is completed in a few minutes. As the amplification is stopped before reaching exponential phase, there is less chance of type competition and diagnostic artifacts. Because samples are individually hybridized and read, there is no signal dilution or competition. In comparison, LA is no longer available. TypeSeq requires library preparation, sample pooling and in-house reagent preparation and extensive QC, and Novaplex (Seegene, Seoul, Korea) requires multiple reactions/sample and 40–50 PCR cycles for detection of individual types [22].

This proof-of-concept study has limitations. Our CodeSets were designed to detect 48 HPV types, but only 8 types were evaluated quantitatively in the defined controls. Further testing with defined controls of more types as well as defined mixtures at differing copy numbers will be required for full quantitative evaluation and exploration of competition between types. The epidemiological samples contained many of the additional types, and performance was evaluated in comparison to LA and TypeSeq assays. It may be noted that evaluation of assay performance using defined samples alone is insufficient since biologic samples that vary in the amount of cellular and viral copies would pose a greater challenge in specific and accurate detection of HPV than purified plasmid. Further testing combining additional defined controls with more epidemiologic samples and lot-to-lot variation in the synthesis of CodeSets will be required to fully evaluate the performance of the NS platform. The success with No-PCR is encouraging and it is possible that further assay refinements could improve the sensitivity so that the HPV NS platform could be truly amplification-free. We conclude that NS technology has promise for the detection and extended typing of HPV for epidemiological studies monitoring the impact of vaccine, type replacement, natural history studies on viral persistence and host-response, HPV type-dependent risk stratification and for mechanistic studies of HPV-associated carcinogenesis. Given the quantitative nature of the NanoString platform, it is possible that this novel HPV assay could be adapted for viral load determination along with type-specific detection. Neither of the comparator assays used in this study are approved for clinical use as the assay was being developed for epidemiologic considerations. Further work using a clinically oriented study design would be required to evaluate whether the detection, typing or quantitative features of a NS HPV assay would have clinical application [32]. This simple and sensitive platform should be applicable for the detection of other viruses and investigating viral pathogenesis with no or minimal cycles of PCR. 

## Figures and Tables

**Table 1:** HPV type-specific concordance between No-PCR and LA.

HPV Types in Analysis	No-PCR Results	LA Results		Total	Agreement (%, k) ^$^	McNemar’s *p*-Value	Positive Agreement (%)	Sensitivity (%)	Specificity (%)
		+	−						
37 Types included in LA	+	138	61	199	91 (1346/1480); k = 0.621 (95% CI 0.562–0.679) (substantial)	0.342	67	65 (138/211)	95.2 (1208/1269)
	−	73	1208	1261					
	Total	211	1269	1480					
14 HR types	+	72	24	96	90.7 (508/560) k = 0.678 (95% CI 0.597–0.760) (substantial)	0.678	73	72 (72/100)	95 (436/460)
	−	28	436	464					
	Total	100	460	560					
HPV16/18 only	+	21	3	24	90 (72/80) k = 0.767 (95% CI 0.615–0.919) (substantial)	0.727	84	81 (21/26)	94 (51/54)
	−	5	51	56					
	Total	26	54	80					

^$^ Agreement (%, k) means percentage of agreement and kappa coefficient (k) with 95% Confidence Interval (CI) and interpretation.

**Table 2:** HPV type-specific concordance between PCR-45 and LA.

HPV Types in Analysis	PCR-45 Results	LA Results		Total	Agreement (%, k) ^$^	McNemar’s *p*-Value	Positive Agreement (%)	Sensitivity (%)	Specificity (%)
		+	−						
All 37 LA types	+	212	9	221	96.2 (1425/1480) k = 0.862 (95% CI 0.828–0.898) (substantial)	<0.000001	89	82 (212/258)	99.3 (1213/1222)
	−	46	1213	1259					
	Total	258	1222	1480					
14 HR types	+	103	1	104	97.1 (544/560) k = 0.91 (95% CI 0.867–0.953) (almost perfect agreement)	<0.000519	93	87.3 (103/118)	99.8 (441/442)
	−	15	441	456					
	Total	118	442	560					
HPV16/18 only	+	25	0	25	100 (80/80) k = 1.00 (95% CI 1.00–1.00) (perfect agreement)	1.00	100	100 (25/25)	100 (55/55)
	−	0	55	55					
	Total	25	55	80					

^$^ Agreement (%, k) means percentage of agreement, and kappa coefficient (k) with 95% Confidence Interval (CI) and interpretation.

**Table 3:** HPV genotype-specific comparison between PCR-15 with LA.

HPV Type *	+/+	+/−	Test Results ** −/+	−/−	Agreement (%)	Positive Agreement (%)	Kappa	95% CI	Interpretation	*p*-Value
All 37 types	272	95	24	2384	95.7	82.1	0.796	0.761–0.832	Substantial	<0.000001
14 HR types	134	34	6	876	96.2	87.0	0.848	0.802–0.894	Almost perfect	<0.0001
LR types	138	61	18	1508	95.4	77.7	0.752	0.700–0.804	Substantial	<0.0000001
HPV16/18	30	1	0	119	99.3	98.4	0.979	0.939–1.00	Almost perfect	1
**16 ^β^**	**16**	**1**	**0**	**58**	**98.7**	**97**	**0.961**	**0.886–1.00**	**Almost perfect**	**1**
**18 ^β^**	**14**	**0**	**0**	**61**	**100**	**100**	**1.000**	**1.000–1.000**	**Perfect**	**1**
**31 ^β^**	**8**	**3**	**1**	**63**	**94.7**	**80.0**	**0.77**	**0.554–0.985**	**Substantial**	**0.617**
**33 ^β^**	**6**	**3**	**0**	**66**	**96**	**80.0**	**0.779**	**0.54–1.00**	**Substantial**	**0.248**
**35**	**7**	**3**	**0**	**65**	**96**	**82.4**	**0.802**	**0.586–1.00**	**Almost perfect**	**0.248**
**39**	**9**	**3**	**1**	**62**	**94.7**	**81.9**	**0.787**	**0.587–0.987**	**Substantial**	**0.617**
**45 ^β^**	**11**	**2**	**0**	**62**	**97.3**	**91.7**	**0.901**	**0.766–1.00**	**Almost perfect**	**0.479**
**51**	**13**	**2**	**0**	**60**	**97.3**	**92.9**	**0.912**	**0.793–1.00**	**Almost perfect**	**0.479**
**52 ^β^**	**15**	**3**	**0**	**57**	**96**	**90.9**	**0.884**	**0.756–1.00**	**Almost perfect**	**0.248**
**56**	**5**	**3**	**1**	**66**	**94.7**	**71.4**	**0.686**	**0.397–0.974**	**Substantial**	**0.617**
**58 ^β^**	**9**	**2**	**1**	**63**	**96**	**85.7**	**0.834**	**0.651–1.00**	**Almost perfect**	**1**
**59**	**11**	**4**	**1**	**59**	**93.3**	**81.5**	**0.775**	**0.587–0.962**	**Substantial**	**0.371**
**66**	**5**	**4**	**0**	**66**	**94.7**	**71.4**	**0.687**	**0.404–0.971**	**Substantial**	**0.133**
**68b**	**5**	**1**	**1**	**68**	**97.3**	**83.3**	**0.819**	**0.574–1.00**	**Almost perfect**	**0.479**
11	4	1	0	70	98.7	88.9	0.882	0.654–1.00	Almost perfect	1
26	4	1	0	70	98.7	88.9	0.882	0.654–1.00	Almost perfect	1
40	4	1	0	70	98.7	88.9	0.882	0.654–1.00	Almost perfect	1
42	11	2	1	61	96	88.0	0.856	0.697–1.00	Almost perfect	1
53	11	2	0	62	97.3	91.7	0.901	0.766–1.00	Almost perfect	0.479
54	7	3	0	65	96	82.4	0.802	0.586–1.00	Substantial	0.248
55	5	4	0	66	94.7	71.4	0.687	0.404–0.971	Substantial	0.133
61	6	2	1	66	96	80.0	0.778	0.536–1.00	Substantial	1
62	13	2	1	59	96	89.7	0.872	0.730–1.00	Almost perfect	1
64	3	4	0	68	94.7	60.0	0.576	0.210–0.942	Moderate	0.133
67	5	4	1	65	93.3	66.7	0.631	0.336–0.926	Substantial	0.371
69	3	3	0	69	96	66.7	0.648	0.282–1.00	Substantial	0.248
6	8	6	0	61	92	72.7	0.684	0.455–0.914	Substantial	0.041
70	6	4	0	65	94.7	75.0	0.722	0.468–0.97	Substantial	0.133
71	3	0	0	72	100	100	1	1.000–1.000	Perfect	1
72	6	1	0	68	98.7	92.3	0.916	0.753–1.00	Almost perfect	1
73	7	1	2	65	96	82.4	0.801	0.583–1.00	Almost perfect	1
81	6	1	0	68	98.7	92.3	0.916	0.753–1.00	Almost perfect	1
82 Subtype IS39	5	8	1	61	88.0	52.6	0.468	0.184–0.752	Moderate	0.045
82	3	5	4	63	88.0	40.0	0.334	0.002–0.669	Fair	1
83	3	1	1	70	97.3	75.0	0.736	0.385–1.00	Substantial	0.479
84	6	5	5	59	86.7	54.5	0.467	0.186–0.748	moderate	0.751
89	9	0	1	65	98.7	94.7	0.94	0.823–1.00	Almost perfect	1

* HPV Type: Only 37 types shared between PCR-15 and LA are included in the analysis. Bold = 14 High-Risk (HR) types; ^β^ = seven nonvalent HR types (16, 18, 31, 33, 45, 52, 58); LR = Low-Risk types. Genotype-sample combination per type = 75; ** Test Results: +/+ (PCR-15^+^/LA^+^), +/− (PCR-15^+^/LA^−^), −/+ (PCR-15^−^/LA^+^) and −/− (PCR-15^−^/LA^−^).

**Table 4:** HPV type-specific concordance between PCR-15 and TypeSeq.

HPV Type *	+/+	+/−	Test Results ** −/+	−/−	Agreement (%)	Positive Ageement (%)	Kappa	95% CI	Interpretation	*p*-Value
All 47 types	218	72	33	1745	94.9	80.6	0.777	0.736–0.888	Substantial	0.000178
14 HR types	108	17	10	481	95.6	88.9	0.862	0.811–0.912	Almost perfect	0.247789
33 LR types	110	55	23	1264	94.6	73.8	0.709	0.648–0.770	Substantial	0.000378
HPV16/18	22	3	0	63	96.6	93.6	0.913	0.817–1.00	Almost perfect	0.248
**16 ^β^**	**13**	**2**	**0**	**29**	**95.5**	**92.9**	**0.895**	**0.755–1.00**	**Almost perfect**	**0.479**
**18 ^β^**	**9**	**1**	**0**	**34**	**97.7**	**94.7**	**0.933**	**0.803–1.00**	**Almost perfect**	**1.000**
**31 ^β^**	**8**	**1**	**0**	**35**	**97.7**	**94.1**	**0.927**	**0.786–1.00**	**Almost perfect**	**1.000**
**33 ^β^**	**5**	**1**	**0**	**38**	**97.7**	**90.9**	**0.896**	**0.696–1.00**	**Almost perfect**	**1.000**
**35**	**7**	**1**	**2**	**34**	**93.2**	**82.4**	**0.781**	**0.545–1.00**	**Substantial**	**1.000**
**39**	**7**	**0**	**0**	**37**	**100.0**	**100.0**	**1.000**	**1.00–1.00**	**Almost perfect**	**1.000**
**45 ^β^**	**7**	**2**	**1**	**34**	**93.2**	**82.4**	**0.781**	**0.545–1.00**	**Substantial**	**1.000**
**51**	**8**	**2**	**0**	**34**	**95.5**	**88.9**	**0.861**	**0.674–1.00**	**Almost perfect**	**0.479**
**52 ^β^**	**14**	**1**	**0**	**29**	**97.7**	**96.6**	**0.949**	**0.849–1.00**	**Almost perfect**	**1.000**
**56**	**3**	**3**	**3**	**35**	**86.4**	**50.0**	**0.421**	**0.037–0.805**	**Moderte**	**0.683**
**58 ^β^**	**8**	**0**	**1**	**35**	**97.7**	**94.1**	**0.927**	**0.786–1.00**	**Almost perfect**	**1.000**
**59**	**11**	**1**	**1**	**31**	**95.5**	**91.7**	**0.885**	**0.730–1.00**	**Almost perfect**	**0.479**
**66**	**4**	**2**	**0**	**38**	**95.5**	**80.0**	**0.776**	**0.479–1.00**	**Substantial**	**0.479**
**68b**	**4**	**0**	**2**	**38**	**95.5**	**80.0**	**0.776**	**0.479–1.00**	**Substantial**	**0.479**
6	8	1	0	35	97.7	94.1	0.927	0.786–1.00	Almost perfect	1.000
11	2	0	0	42	100.0	100.0	1.000	1.00–1.00	Almost perfect	1.000
*13*	*0*	*2*	*0*	*42*	*95.5*	*0.0*	*0.000*	*0*	*Poor*	*0.479*
26	2	0	0	42	100.0	100.0	1.000	1.00–1.00	Almost perfect	1.000
*30*	*4*	*1*	*1*	*38*	*95.5*	*80.0*	*0.774*	*0.474–1.00*	*Substantial*	*0.479*
40	1	1	1	41	95.5	50.0	0.476	0.143–1.00	Moderate	0.479
42	7	0	2	35	95.5	87.5	0.848	0.644–1.00	Almost perfect	0.479
*43*	*1*	*0*	*0*	*43*	*100*	*100.0*	*1.000*	*1.00–1.00*	*Almost perfect*	*1.000*
*44*	*3*	*2*	*1*	*38*	*93.2*	*66.7*	*0.629*	*0.245–1.00*	*Substantial*	*1.000*
53	10	2	1	31	93.2	87.0	0.824	0.632–1.00	Almost perfect	1.000
54	4	3	0	37	93.2	72.7	0.692	0.371–1.00	Substantial	0.248
55	3	2	0	39	95.5	75.0	0.727	0.371–1.00	Substantial	0.479
61	3	3	0	38	93.2	66.7	0.633	0.261–1.00	Substantial	0.248
62	10	1	0	33	97.7	95.2	0.938	0.817–1.00	Almost perfect	1.000
64	3	2	2	37	90.9	60.0	0.549	0.156–0.941	Moderte	0.617
67	5	0	1	38	97.7	90.9	0.896	0.696–1.00	Almost perfect	1.000
*68a*	*0*	*8*	*0*	*36*	*81.8*	*0.0*	*0.000*	*0*	*Poor*	*0.013*
69	1	2	0	41	95.5	50.0	0.482	0.117–1.00	Moderte	0.479
70	2	3	0	39	93.2	57.1	0.542	0.097–0.987	Moderte	0.248
71	2	0	0	42	100	100.0	1.000	1.00–1.00	Almost perfect	1.000
72	3	1	0	40	97.7	85.7	0.845	0.549–1.00	Almost perfect	1.000
73	5	1	1	37	95.5	83.3	0.807	0.548–1.00	Almost perfect	0.479
*74*	*5*	*0*	*4*	*35*	*90.9*	*71.4*	*0.665*	*0.371–0.960*	*Moderate*	*0.133*
81	5	0	0	39	100.0	100.0	1.000	1.00–1.00	Almost perfect	1.000
82 Subtype IS39	2	3	1	38	90.9	50.0	0.453	0.008–0.899	Moderate	0.617
82	3	4	1	36	88.6	54.5	0.486	0.107–0.865	Moderate	0.371
83	0	2	1	41	93.2	0.0	0	0	Poor	1.000
84	4	4	1	35	88.6	61.5	0.553	0.211–0.894	Moderate	0.371
*87*	*2*	*2*	*1*	*39*	*93.2*	*57.1*	*0.535*	*0.073–0.998*	*Moderate*	*1.000*
89	5	1	2	36	93.2	76.9	0.73	0.440–1.00	Substantial	1.000
*90*	*3*	*1*	*0*	*40*	*97.7*	*85.7*	*0.845*	*0.549–1.00*	*Almost perfect*	*1.000*
*91*	*2*	*0*	*2*	*40*	*95.5*	*66.7*	*0.645*	*0.196–1.00*	*Substantial*	*0.479*
*114*	*0*	*3*	*0*	*41*	*93.2*	*0.0*	*0.000*	*0*	*Poor*	*0.248*

* HPV Type: 47 types shared between PCR-15 and TypeSeq are included in the analysis. Bold = High-Risk (HR) types; ^β^ = seven nonvalent HR types (16, 18, 31, 33, 45, 52, 58); LR = Low-Risk types. Italics indicates 10 HPV types (HPV13, 30, 43, 44, 68a, 74, 87, 90, 91, 114) shared by PCR-15 and TypeSeq but not in LA. Genotype-sample combination per type = 44; ** Test Results: +/+ (PCR-15^+^/TypeSeq^+^), +/− (PCR-15^+^/TypeSeq^−^), −/+ (PCR-15^−^/TypeSeq^+^) and −/− (PCR-15^−^/TypeSeq^−^).

## Data Availability

The data presented in this study are available upon request from the corresponding author. The data are not publicly available due to technological considerations.

## Supplementary Materials

The following are available online at https://www.mdpi.com/1999-4915/13/2/188/s1: Table S1: HPV type-specific capture and reporter sequences designed within the L1 gene for testing by No-PCR (Version 1). Table S2: HPV type-specific capture and reporter sequences designed within the E6 gene for testing with No-PCR (Version). Table S3: HPV type-specific capture and reporter probes within the 450 bp L1 region amplified by consensus primers (Version 2). Table S4: Details on samples tested with No-PCR. Table S5: Details of samples tested with PCR-15 and HPV TypeSeq. Table S6: L1 consensus primer targeting 450 bp PCR product. Table S7: Type-specific concordance between results for E6 and L1 CodeSets for direct NanoString testing (No-PCR assay). Table S8: Type-specific concordance of HPV CodeSets in comparison to LA or TypeSeq (Z-test results) under different testing and sample conditions.
