# Supplementary material for: NanoString Technology for Human Papillomavirus Typing
Source: Viruses. 2021 Jan 27;13(2):188. doi: 10.3390/v13020188 (PMC7911781; doi:10.3390/v13020188)
Supplement: Supplementary file 1 [file viruses-13-00188-s001.pdf]

Table S1. HPV type-specific capture and reporter sequences designed within the L1 gene for testing by No-PCR (Version 1)

HPV type	Targeted genomic region	Capture (Cs) and Reporter (Rs) sequences (5′ → 3′)
6	6750-6849	Cs- GTTGGTACTGCGTGTGGTATCTACCACAGTAACAAACAGTTGATTACCCC<br>Rs- TCAGAATTGGTGTATGTGGAAGATGTAGTTACGGATGCACATAATGTCAT
11	6735-6834	Cs- ATTTGTACTGCGTGTGGTATCTACCACAGTAACAAACAAGTGGTTTCCCC<br>Rs- TCTGAATTAGTGTATGTAGCAGATTTAGACACAGATGCACATAGTGTCAT
13	7132-7231	Cs- TTTCCTACCTACACGAATAGGGGACCTAGACTGAACGCCTGTTTGTAATA<br>Rs- TTAGCTTTTTTACGTGTAGTAGGTGTGGCAGTAGATGTAGATGCAGCACG
16	6209-6308	Cs- GCACCAAAGCCAGTATCAACCATATCACCATCCTGAATAACTGTGTTTAT<br>Rs- TATCCAGTGGAACTTCACTTTTGTTAGCCTGTAATGTAGTAAAGTCCATA
18	6586-6685	Cs- ATTGGTACTGGGAGTGGTATCTACCACAGTAACAAATAATTGATTATGCC<br>Rs- GCATCATATTGCCCAGGTACAGGAGACTGTGTAGAAGCACATATTGTTAA
26	6561-6660	Cs- GTTAGTACTGCGGGTGGTATCAACACAGGTAACAAACAATTGATTGCCCC<br>Rs- GGTTTAAATGGAGTGGATGCAGATGCTGCAGATAATGTACTAATGGTAAG
30	6043-6142	Cs- AATGTTGTCCCTACTATCCTCTGCTGTATCCTGATTAGCTATAGTGGAAC<br>Rs- GCAGGAGTGCACCCAATAATACACAATTGGGTTTGCTTTGGATCAACAGA
31	6707-6806	Cs- TCTTCCAAAATAGCAGGATTCATACTGTGAATATATGTCATTATGTCTGC<br>Rs- AGGTATCCTCCAAAGAACCTGAGGGAGGTGTGGTCAATCCAAAATTCCAA
33	6567-6666	Cs- ATTAGTACTGCGAGTGGTATCTACCACAGTAACAAATACCTGATTGCCCC<br>Rs- TTTTCATTTTTATATGTACTGTCACTAGTTACTTGTGTGCATAAAGTCAT
34	5820-5919	Cs- AAAGGATTACCACTAGTACCTATACCTAATGGCTGTCCGCGTCCAACCTC<br>Rs- TTCCACCAATATATTTTGCAGCATTTTCAGTATCCTCAAGTTTATTCATA
35	6568-6667	Cs- ATTTGTACTACGGGTTGTATCAACTACAGTAACAAACAATTGGTTACTCC<br>Rs- TCATTTTTATATGTACTGTCACTAGAAGACACAGCAGAACACACAGACAT
39	6866-6965	Cs- CTGCAGACTGTAGGTATCTGTAAGTGTCTACCAAACTGGCAGATGGTGGA<br>Rs- ATATGGATCTTTCTTTTCAGGTGCTGGAGCATCCTTTTGACATGTAATGG
40	6927-7026	Cs- CCTCCAACAACGTAGGATCCATTGCATGAATATATGTCATAACCTCTGCA<br>Rs- TGTATCCTCTAAGGATGCAGAGGCTGGAGGAGCAATTTTAAAGTTCCAAT
42	6810-6909	Cs- GTTAGTACTACGGGTAGTATCAACCACAGTTAAAAATAGCTGATTTCCCC<br>Rs- TTAGCAGCTGTATATGTATCACCAGATGTTGCAGTGGCACACAAAGTCAT
43	6724-6823	Cs- GTTTGTACTACGAGTGGTATCTACCACTGTAACAAACAACTGATTCCCAA<br>Rs- TTGTCATATGTACTGGGCACAGTAGGGTCAGTAGAGGCACATAACGTTAA
44	6655-6754	Cs- GTTTGTACTACGGGTAGTATCTACAACAGTAACAAATAACTGATTTCCCC<br>Rs- CTAGTATATGTAGACGGAGGGGACTGTGTAGTGGCAGCACATATTGTCAT
45	6590-6689	Cs- ATTAGTACTGCGGGTAGTGTCCACTACAGTAACAAACAACTGATTATGCC<br>Rs- GGGTCATATGTACTTGGCACAGGATTTTGTGTAGAGGCACATAATGTTAA
51	6494-6593	Cs- ATTTGTACTTCTGGTAGTATCAACACAGGTAATAAAAAGCTGATTGTTCC<br>Rs- CTTGGAGTAAATGTTGGGGAAACCGCAGCAGTGGCAGTGCTAATAGTTAA
52	6882-6981	Cs- ATAGCAGTAGAAGTGACAAATCTGTATGTGTCCTCCAAAGATGCAGACGG<br>Rs- CCTTTAAAGGATCTTCCTTTCCTTTAGGTGGTGTGTTTTTTTGACAAGTT
53	6625-6724	Cs- GTTTGTATTCCTGGTGGTATCCACAACAGTTACAAATAACTGATTGTTCC<br>Rs- TGCTTTGAATTATATGTAGACATAGACTGTGTGGTTGCGGAAAGAGTCAT
54	6313-6412	Cs- TGTTCCCGCCTTAAGTAAAAAAATAAACTGTCCCCATATGCCTCTGCAGC<br>Rs- CAGGCTCACCCATGGTACCTGCCCTATTTAACATATGCCTAACAAACATT
55[&]	7082-7181	Cs- CCACACGAACAGAGGACCGGGCCTGCACACCCGTTTGCAATAAAAATTTC<br>Rs- TTTGGGTTTACTGGAGGATGAAGTGGCTGCAGATGCAGGACGTTTTCTAC
56	6568-6667	Cs- GTTAGTACTTCTAGTAGTATCTACTACAGTAACAAATAATTGATTACCCC<br>Rs- TTTCGTGCATCATATTTACTTAACTGTTCTGTAGCAGTACTAATAGTCAT
58	6616-6715	Cs- ATTAGTGCTACGAGTGGTATCAACCACGGTAACAAATAACTGATTGCCCC<br>Rs- TTATCATTTTTATATGTACCTTCCTTAGTTACTTCAGTGCATAATGTCAT
59	6833-6932	Cs- GCAGCAGATTGAACAAAACGGTATGTGTCAACTAAACTAGCAGTAGGAGG<br>Rs- CATAAGGGTCCTGTTTAACTGGCGGTGCGGTGTCCTTTTGACAAGTTACA
61	7007-7106	Cs- CTTCTGACATGTAATAGCTCTGGACTGCAAAAACCTATATGTGTCTTCTA<br>Rs- AAGGATAACTTGGCATAGCGATCCTCCTTGGGCGGCGGGGCAGCAGCACC
62	7034-7133	Cs- GACGGGGAAGCAGCCCCCTTTTGACATGTAATAGCCCGAGACTGCAAATA<br>Rs- TAAGATCCACAGTCCAAAATGTCATTTGCGCATACGGGTCCACCTTGGGG
64[$]	1-100	Cs- GTTAGAAACAATTGATTATGCCAACAAATTCCATTCTTATGTCCCTGTGC<br>Rs- GTGTGCCTACACAAACAGAAAAGTTTGTACTTCTGGTAGTATCAACAACA
66	6617-6716	Cs- GTTGGTGCTTCTGGTAGTATCCACAACAGTAACAAATACCTGATTACCCC<br>Rs- TCACGGGCATCATATTTAGTTAATGTGCTTTTAGCTGCATTAATAGTCAT
67	6771-6870	Cs- CAGTCCTCTAATATATCTGGATTCATGGTGTGTATGTATTGCATAACATT<br>Rs- TATATGTGTCCTGTAAATTACCTGAAGGAGGTGGTGTAAGGCCAAATTGC
68	6461-6560	Cs- ATTAGTACTGCGCGTTGTATCCACAACGGTAAGAAATAATTGATTATGCC<br>Rs- GAATCATACACAGCTGGTACAGTAGAGTCTGTAGTAGTGGACAATGTAAA

68b[%]	6755-6854	Cs- CGTCTTTTTGACATGTAATTGCTGCTGATTGCAGATAGCGGTATGTATCT<br>Rs- ATTCCAAAAGTTTAAGCCATCATATGGATCCTTTTTAGTAGGTGCAGGGG
69	6517-6616	Cs- GTTGGTACTGCGGGTAGTATCTACACAAGTAACAAACAATTGGTTGCCCC<br>Rs- GGTTTAAAAGTGGCAGATGCAGATTGTGCAGATACAGTACTAATAGTGAG
70	6557-6656	Cs- ATTAGTACTACGTGTAGTGTCCACCACAGTAATAAACAACTGGTTATGCC<br>Rs- GGGCTATATACAGCAGGTATGGCCGTTTCGGTGCAGGCAGACAATGTAAA
71	6819-6918	Cs- ATTTGTACTACGTGATGTGTCCACAACTGTTACAAAAAGCAGATTGCCCC<br>Rs- CTAGAGGCTTTATATGTAGACTCAACAGTTTTGGTAGCACAGATGGACAT
72	7043-7142	Cs- GGCAGCCCCCTTTTGACAGGTAATGGCACGAGACTGCAAAAACCTATAGG<br>Rs- TCCAAAAGGATAAGTTAGCATATGGATCTTCTTTAGGAGGAGGGGT
73	6714-6813	Cs- GTGATGTTACATATCTATATGTTTCCTCTAAAGTACCTGACGGTGGTGGG<br>Rs- TGGGTCCTCTGTTTCTTTAGGAGGTTGAGGACGTTGGCAACTAATAGCCT
74	5811-5910	Cs- GGGGTCAAATAACGAAGTGTCAGGTAAAGCAAATTTATTGGGATCTGGCA<br>Rs- CCCCTACCTACCTCCAAACCCGTACAGGCCCATACCAAACGTTGACTGGA
81	6837-6936	Cs- ATTGGTGCTTCTGGTAGTATCCACCACTGTAACAAACATTTCATTAAACC<br>Rs- TTAGAGGCCTTGTATTCTGCAGCAGCAGATGTAGCTGTGCAAATAGTAAA
82	6912-7011	Cs- TGCAAACTGATCCAAATCCAAAGAAAAGCGTTCCTTAAGGTCTACATTCC<br>Rs- GGTCTGGGTTTGCGTTGGGCACCGATTTGTAATAAAAACTTGCGACCCAA
82_ IS39*	6811-6910	Cs- GCAAGCTGATCTAAATCTAAAGAAAAGCGTTCCTTAAGGTCCACGGTCCA<br>Rs- GCCTGGCCTTGCGTTGTGCACCAATTTGTAATAGGAACTTGCGACCCAAT
83	6969-7068	Cs- GCACGGGACTGCAGATAGCGATAGGTATCATCAAGGCTGGTGGAGGGAG<br>Rs- CTTTTTAGGGGCAGGGGCGGAAGGACCCTTTTGGCAGGTAATA
84	6647-6746	Cs- ATTGGTGCTGCGGGTGGTATCTACCACCGTGACAAACAATTGATTAAACC<br>Rs- TTGGTAGGTTTATATTCTGATTCGGTGTTGGTAGCAGCACTAATAGTAAA
87	6200-6299	Cs- GGCTTACAGCCTACAATTAACAACTGGGTTTGCTTGTAGTCCACAGACAC<br>Rs- GCTGTACATTGGCACAAACAGTACCCTTACTCCAATGCTCCCCTATAGAG
89	6516-6615	Cs- GGTCCTCACCCATAACACCCTGCCTATTAAAAAAGTGGCGAGCAAACATT<br>Rs- TGTATTTCTGTTATTAGCAGCAGACCCAGGCATATACAAGGTTTTTGGCA
90	6774-6873	Cs- ATTGGTGCTACGTGTAGTATCAACCACAGTTACAAAAAGCTGATTACCCC<br>Rs- TTGGAAGCCTTGTATGTGTCAGAGGGTGTTTGTGTGGCACAAATAGTCAT
91	6838-6937	Cs- GTTAGTGCTGCGAGTTGTATCCACAACAGTTACAAACACCTGGTTACCAA<br>Rs- TTGTCATATGTAGTAGGTAGCACAGACTCAGTGGATGCACACAAGGTTAA
114	6368-6467	Cs- CCAATGCTCTCCAATGGAAGGCTTACAGCCCACAATTAAAAGCTGTGTCT<br>Rs- GGCGTTGTTGGACATTGGAGCATACAGTGCCCTTACC
HBB-1**	2143-2242	Cs- CCCAGTTTCTATTGGTCTCCTTAAACCTGTCTTGTAACCTTGATACCAAC<br>Rs- AGAGAGTCAGTGCCTATCAGAAACCCAAGAGTCTTCTCTGTCTCCACATG
HBB-2**	3256-3355	Cs- AGGGCCTAGCTTGGACTCAGAATAATCCAGCCTTATCCCAACCATAAAAT<br>Rs- TGCCCAGGAGCTGTGGGAGGAAGATAAGAGGTATGAACATGATTAGCAAA

[&]Reference sequence: GeneBank accession No.: U31791.1

[$]Reference sequence: GenBank accession No.: AJ812226.1

[%]Reference sequence: GenBank accession No.: FR751039.1

*Reference sequence: GeneBank accession No.: AF293961.1

**Reference sequence: GeneBank accession No.: GU324922.1

Table S2. HPV type-specific capture and reporter sequences designed within the E6 gene for testing with No-PCR (Version)

HPV type	Targeted genomic region	Capture (Cs) and Reporter (Rs) sequences (5′ → 3′)
6	375-474	Cs- TGACACAGGTAGCACCGAATTAGCACGTCTAAGATGTCTTGTTTAGTTTC<br>Rs- GCGCCTTGGTTAGTATATGTTTTACCTTTTCTACTTCACACAGCGGTTTG
11	232-330	Cs- GGAAAGTTGTCTCGCCACACAACCTTTAGGTTCTTATAGGCATATGCATA<br>Rs- ATTGGTTAATTTTCCCTTGCAGTTCTAAGCAACAGGCACACGCTGCAAAG
13	287-386	Cs- CTAAACTGGTTAATCTTTCCTTGTATTTCTAAGCAGCATGCACAAGCCGC<br>Rs- GCTTTGTGTCTTCTTCAACTGTTACAGCAAATCCCGCGAAGTCAAAATGC
16	448-547	Cs- ACCGACCCCTTATATTATGGAATCTTTGCTTTTTGTCCAGATGTCTTTGC<br>Rs- TTCTCTACGTGTTCTTGATGATCTGCAACAAGACATACATCGACCGGTCC
18	467-566	Cs- ACTGGCCTCTATAGTGCCCAGCTATGTTGTGAAATCGTCGTTTTTCATTA<br>Rs- TCGTTCCTGTCGTGCTCGGTTGCAGCACGAATGGC
26	412-511	Cs- TTTTCATCCACAATTCTTTGTTTTTCTTCTGGCCCCAATGGCATTTGACA<br>Rs- AATTTGTACACAACCCTTTCCACTGCCCTGCTATTTCGTGAAATCGTCGC
30	193-292	Cs- ATTATATACCTCTGAGCTGGATAATTCCTTCTTGCAATATACACACTGTA<br>Rs- GCATATGGGCTGTCCTCCCTATATACCAGTCTTAAATCTTTACATGCAAA
31	449-548	Cs- TTCCTCCTATGTTGTGGAATCGTTTCTTTTTATCCAAATGTCTTTGTTTT<br>Rs- TACGAGGTCTTCTCCAACATGCTATGCAACGTCCTGTCCACC
33	111-210	Cs-CTTGGCACAAATCATGCAATGTTCGTGGTTTTTCCTCAGTGTCTTGAAAC<br>Rs- TTTGCATTCCACGCACTGTAGTTCAATGTTGTGTATAGTTGTCTCCAATG
34	265-364	Cs- Cs- GTAAAACAGTAAACACGGTTGACATACCCCAAGTGGTTTCCCCTTTCTAT<br>Rs- GTCCGTCCATACACTGATTGGTTATATCTTCTATATTGTCTAACCTTTGA
35	453-552	Cs-CCGTCCACCGATGTTATGGAATCGTTTTTTTTCTTCTAAATGTCTTTGCT<br>Rs- GTTTCTCTACGTGTTGGTTTCCAACAGGACATACACCGACCTGTCCA
39	198-297	Cs- AAATTCATATACCTCGGTTTGCTGTAGTGGTCGTCTGCAATAGACACAGG<br>Rs- GCAGCTAGTGGTTCCCCGTCCCTATATACTACATATAAATCACTAAATGC
40	256-355	Cs- GTCCAGGCACCGTGGACATGCGGCGTGTGGAAAGTCG<br>Rs- GCATAGGCTGCGTATCTAAAGTTTCTGTATTGGTTTACTTTTCCGTGCAG
42	214-313	Cs- ATCTTTAAAATGGTACGCGAGCACCTCTGCGCCTGTTAAGTGCTTTTTGC<br>Rs- CAAAATGCACATGCAGCATATGGAAAGTCCTTCCTCCACACCACTACCAA
43	324-423	Cs- TCTTCTACAGTATCTGCATATGCTGCGTAGTCAAAGTGCCTATATTGACT<br>Rs- GGCACTTACAGCATCTAATGCACAAATCAAACACTGTTTGCTTAGTTTCT
44	438-537	Cs- TTAATGAATCGCGCCTTGTCCAATATGTGGCGCACCTTTTCCACGTGGCA<br>Rs- TGCATGATGTCCAACAATGGAAGCAGCGACCCTTCCAGGTATCTTGTAAT
45	464-563	Cs- ACTGCCCTCGGTACTGTCCAGCTATGCTGTGAAATCTTCGTTTGT<br>Rs- CCTACGTCTGCGAAGTCTTTCTTGCCGTGCCTGGTCACAACATGTATTAC
51	431-530	Cs- TATTTCATGGAACCTTTTTTTTTCGTCCACCAATTTTTGCTTTTCTTCAG<br>Rs- CAGCAATTAGCGCATTGCCCCGTCCAACGTCCCGC
52	147-246	Cs- TGCACACACTGCAGCCTTATTTCATGCACCGATTCTTCCAGC<br>Rs- CTGTAAATAGAAACTTGTATACCTCTCTTCGTTGTAGCTCTTTTTTGCAC
53	162-261	Cs- CAGAACACACAGCCAAGTTGCAGCTCCAGCAATGGTTTATTCACAACTTC<br>Rs- GATCTGTATATGCAAAATTATATACCTCTGACGCTGTCAATGCCTTCTTG
54	262-361	Cs- TTCTAGGCACAGTGCACATGCAGCATGTGGAAAGCCGTGTCT<br>Rs- CACAGGCACGCACGGTCGCGATGCCTTCTATAATTTATTTGCCCGTGCAG
55[&]	287-386	Cs- GCCTAAATTGATTGACCTTACCTTGTAGTTCTAAACAAATGGCACAGGCT<br>Rs- CTGCTTTGTCTCTTCTTCCACTGTTAATGCATATCCCGCGAAGTCAAAAT
56	409-508	Cs- TTGCTTTTCCTCCGGAGTTAACGGACTTTGACATCTGTAGCACCTTATTA<br>Rs- GTCCAACCATGTGCTATTAGATGAAATCGTCTTTTTCTGTCACAATGCAA
58	128-227	Cs- ACAGATGTCTCCAACGCCTGACACAAATCATGCAATGTCCGTGGTTTCTC<br>Rs- ATCGCTGCAAAGTCTTTTTGCATTCAACGCATTTCAATTCGATTTCATGC
59	301-400	Cs- TGTAACGGTGTCTTGGTTTCAGCCTCTAATGTTTCTCCATACACGGAATC<br>Rs- CTGTTGGACATAGAGGTTTTAGGCATCTATAACAGCGTATCAGCAGCTCA
61	224-323	Cs- CAAATGGCCAGTTATGTCTCCACACAATGCTTAGCTCCTTTAGTGCAAAC<br>Rs- CCCTCACTTTTACTTCACGTGCCAAGCATGGTGCGCATACTC
62	179-278	Cs- CCGTAGCTCTCTTACTTTAATCTCTCTCAATAGGCATGCTGCGCATACTC<br>Rs- CTGTTTCCTCTTCTACTGTTATTCCAAAGCATGAGTGCTGCCACCG
66	461-560	Cs- CGGTCCATGCATATGCTATATAATGAAATCGTCTTTTATGTTCACAGTGC<br>Rs- AGATTCTGTAGCTTGTCTACTCGTATGTCTCCAACACTGCAAACATGACC
67	136-235	Cs- CAAACTTATTTCATGCACCGTGGTTTCCAAAGCTTCACACAATTCGTGCA<br>Rs- AAGTCATATACCTCGTTTCTGTCCAAAGTTTTTTTGCACTGCACGCAGGG
68	95-194	Cs- GGCAAATTCATATACCTCTGTCCGTTGTAGTTGCCTTCTGCAATAGACAC<br>Rs- CATGCAGCAAATGGTACCCCGTCTCTATACACTACACATAGGTCACTAAA
68b[$]	5183-5282	Cs- AATGTAGCATTATGGAATGCAGTATCCAATACTGTAGTATTGTCAGTATC<br>Rs- TAGATGCTGTAGACGCTAATGAAGGAACAGATATATGGGAACGGGAGGTA
69	201-300	Cs- AAATCACATATTGCAAAGTTATATACATCTGCCCATTCTAATGTTTTCTT<br>Rs- TACATTTTTTACATGCACCATATGCACTATCATTTCTATACACTATTCTT

70	295-394	Cs- GCCGTAGTTCCCTTACTTTAGCATGAAATTTAATACATTTTTGGCATGCA<br>Rs- GGTATTAGTTATGCTTTCCAAAGTTGTTGCATACACCGAGTTCGAATAAT
71	228-327	Cs- ACTTTCAGCTCCTTATATGCAAAGGCCACGACTTCCACGTCTGTTAATTG<br>Rs- TTTCCAAACAGCAGGCACATGCAGCAAACGGAAAACCACTTCTCCACACA
72	305-404	Cs- GTCCGTAGCCCGAATACGTCCAGTATCGTAGCTCC<br>Rs- CCTTATATATAGTTCTGCTAATGATTTGCCTGTTTCCTGTTCCACAGTGG
73	117-216	Cs- ATATTCACTTCGTCACATAACGCTTGTAGCTTGTATGGTCGTTCTTCTGA<br>Rs- ACAGTCCACGTTGGCAAAACACACAGTCCAGGTTTATATCATGTATAGAA
74	261-360	Cs- AGCGTATTAGCACATCCAAAATTGACTGCTTTGTTTCTTCTTCCACTGTT<br>Rs- TTCCACGTGGCACAGCGGCTTATGGCACAGGTAGC
81	430-529	Cs- GCCTGTCACAAGATACTCCTTTTCTTGGTATGTTAACGGTTTATAGCATG<br>Rs- TGGCAGCACTTTCCCATCCACTCTCCAGCTATTTTATGAAAGTGGATTAG
82*	686-785	Cs- AATTCTGTAACACGTATCCTGTCCAGCTTGTCTGGCTGGCTG<br>Rs- CTTTCCACTGCGAGCTGTACAACACTCGAACACCTGCAACAGTGCACTTT
83	319-318	Cs- TTTCTTCTTCTACTGTTGCCCCGTAGCTTGAGTGG<br>Rs- CTTCAGGCACATATGGCATCGTATATACAGCTGTGCCAGTGGTGTTTTTG
84	154-253	Cs- CTGCATAAGCATTTCATGCACACCCCATGAGGGAAATTATACCTCCAAAC<br>Rs- TAATCCCAGTGGCGTAGCTCACGTACTTTGGCTTCC
82_ IS39**	366-465	Cs- AATTTGCACACTGTCCCGTCCAGCGTCCCGCTATTTCATGAAACCTTTTT<br>Rs- CTGGGTTTCGCTACGTTGTCTTGCCGCTGTTCTGC
87	243-342	Cs- AGGCACACTCCATAAGGAAATCCAAATCTCCACACCAGCAATAATTCCCT<br>Rs- ACTCCCAGTGACGAAGCTCACGTTTCTTGGCTTCTCTGAATAGGCATTGT
89	358-453	Cs- TTGCAACACTTTCCTGTCCACTCTCCAGATATTTTATGAAATCGCTCGTT<br>Rs- ATGGTCGGCGTGCGGCCGTGCATATTACCCTGCAG
90	153-252	Cs- CTTCCAGACACGCAGGACATGCTGCAAATGGAAAATGCTTTCTCCACACA<br>Rs- TGCATAGCTAGAATACTGCCAGTATCTAAATTGCCTTAGTTTCCCGGCTG
91	400-499	Cs- TCCTCTACTGTTACTGCTAATGCCGCGTACTTAAAATGCCTATATTGGCT<br>Rs- GGCACTTACAACAGCGAATGCACAACTCGAATACAGTTTGTTGTGTTTCT
114	496-595	Cs- ATAGCACAACGGCTTGCAACACGCGTGGCACCTTATATATATTTGTGCAA<br>Rs- GATATTTTGTAAAAATGCATGCGCGATTCCACCATATGCTCCTTTTCCTG

[&]Reference sequence: GeneBank accession No.: U31791.1

[$]Probes were designed based on L2. Reference sequence: GeneBank accession No.: FR751039.1

*Probes were designed based on E7

**Reference sequence: GeneBank accession No.: AF293961.1

Table S3. HPV type-specific capture and reporter probes within the 450 bp L1 region amplified by consensus primers (Version 2).

HPV type	Targeted genomic region	Capture (Cs) and Reporter (Rs) sequences (5′ → 3′)
6	6750-6849	Cs- GTTGGTACTGCGTGTGGTATCTACCACAGTAACAAACAGTTGATTACCCC<br>Rs- CAGAATTGGTGTATGTGGAAGATGTAGTTACGGATGCACATAATGTCAT
11	6735-6834	Cs- ATTTGTACTGCGTGTGGTATCTACCACAGTAACAAACAAGTGGTTTCCCC<br>Rs- TCTGAATTAGTGTATGTAGCAGATTTAGACACAGATGCACATAGTGTCAT
13	6710-6809	Cs- CACACACAGTCATGTTAGTACTGCGTGTAGTATCAACTACAGTAACAAAC<br>Rs- TTTATATTCTGTGGCCTTATATGTGTCTGAAAGAGATGATGTAGTGGCTG
16	6883-6982	Cs- TATGTTTTTGACAAGCAATTGCCTGGGATGTTACAAACCTATAAGTATCT<br>Rs- TTCCCAAAAAGTGTATTTTTTAAGGGGATCTTCTTTAGGTGCTGGAGGTG
18	6586-6685	Cs- ATTGGTACTGGGAGTGGTATCTACCACAGTAACAAATAATTGATTATGCC<br>Rs- GCATCATATTGCCCAGGTACAGGAGACTGTGTAGAAGCACATATTGTTAA
26	6561-6660	Cs- GTTAGTACTGCGGGTGGTATCAACACAGGTAACAAACAATTGATTGCCCC<br>Rs- GGTTTAAATGGAGTGGATGCAGATGCTGCAGATAATGTACTAATGGTAAG
30	6619-6718	Cs- GTTCCTAGTGGTGTCCACAACAGTAACAAATACCTGGTTGCCC<br>Rs- GAATTATATGTGGATAACGTTTGTGTGGTTGCAGATATAGTCATGTTTGT
31	6707-6806	Cs- TCTTCCAAAATAGCAGGATTCATACTGTGAATATATGTCATTATGTCTGC<br>Rs- AGGTATCCTCCAAAGAACCTGAGGGAGGTGTGGTCAATCCAAAATTCCAA
33	6567-6666	Cs- ATTAGTACTGCGAGTGGTATCTACCACAGTAACAAATACCTGATTGCCCC<br>Rs- TTTTCATTTTTATATGTACTGTCACTAGTTACTTGTGTGCATAAAGTCAT
34	6701-6800	Cs- CTTCTAAAGTACCTGAAGGCGGTGGTGTAAGACCAAAATTCCACTGTTCC<br>Rs- TTGCGGACGCTGACATGTAATGGCCTGTGAAGTAACATATCTATATGTTT
35	6568-6667	Cs- ATTTGTACTACGGGTTGTATCAACTACAGTAACAAACAATTGGTTACTCC<br>Rs- TCATTTTTATATGTACTGTCACTAGAAGACACAGCAGAACACACAGACAT
39	6866-6965	Cs- CTGCAGACTGTAGGTATCTGTAAGTGTCTACCAAACTGGCAGATGGTGGA<br>Rs- ATATGGATCTTTCTTTTCAGGTGCTGGAGCATCCTTTTGACATGTAATGG
40	6927-7026	Cs- CCTCCAACAACGTAGGATCCATTGCATGAATATATGTCATAACCTCTGCA<br>Rs- TGTATCCTCTAAGGATGCAGAGGCTGGAGGAGCAATTTTAAAGTTCCAAT
42	6810-6909	Cs- GTTAGTACTACGGGTAGTATCAACCACAGTTAAAAATAGCTGATTTCCCC<br>Rs- TTAGCAGCTGTATATGTATCACCAGATGTTGCAGTGGCACACAAAGTCAT
43	6724-6823	Cs- GTTTGTACTACGAGTGGTATCTACCACTGTAACAAACAACTGATTCCCAA<br>Rs- TTGTCATATGTACTGGGCACAGTAGGGTCAGTAGAGGCACATAACGTTAA
44	6655-6754	Cs- GTTTGTACTACGGGTAGTATCTACAACAGTAACAAATAACTGATTTCCCC<br>Rs- CTAGTATATGTAGACGGAGGGGACTGTGTAGTGGCAGCACATATTGTCAT
45	6590-6689	Cs- ATTAGTACTGCGGGTAGTGTCCACTACAGTAACAAACAACTGATTATGCC<br>Rs- GGGTCATATGTACTTGGCACAGGATTTTGTGTAGAGGCACATAATGTTAA
51	6494-6593	Cs- ATTTGTACTTCTGGTAGTATCAACACAGGTAATAAAAAGCTGATTGTTCC<br>Rs- CTTGGAGTAAATGTTGGGGAAACCGCAGCAGTGGCAGTGCTAATAGTTAA
52	6882-6981	Cs- ATAGCAGTAGAAGTGACAAATCTGTATGTGTCCTCCAAAGATGCAGACGG<br>Rs- CCTTTAAAGGATCTTCCTTTCCTTTAGGTGGTGTGTTTTTTTGACAAGTT
53	6625-6724	Cs- GTTTGTATTCCTGGTGGTATCCACAACAGTTACAAATAACTGATTGTTCC<br>Rs- TGCTTTGAATTATATGTAGACATAGACTGTGTGGTTGCGGAAAGAGTCAT
54	6703-6802	Cs- ACATCTGCTGTAAGGGTTATGGTACATAACTGAAATATAAACTGTAAATC<br>Rs- AGTTCCAGTCCTCTAGAATAGTGGGATTCATTCCATGAATATAGGCCATA
55[&]	6680-6779	Cs- GAGTTGTAGCAGCACATATTGTCATGTTTGTACTACGTGTAGTATCTACA<br>Rs- TCGCATGTATTGTTTATATTCTGTACTATTATATGTTGTAGATGGAGACT
56	6568-6667	Cs- GTTAGTACTTCTAGTAGTATCTACTACAGTAACAAATAATTGATTACCCC<br>Rs- TTTCGTGCATCATATTTACTTAACTGTTCTGTAGCAGTACTAATAGTCAT
58	6616-6715	Cs- ATTAGTGCTACGAGTGGTATCAACCACGGTAACAAATAACTGATTGCCCC<br>Rs- TTATCATTTTTATATGTACCTTCCTTAGTTACTTCAGTGCATAATGTCAT
59	6833-6932	Cs- GCAGCAGATTGAACAAAACGGTATGTGTCAACTAAACTAGCAGTAGGAGG<br>Rs- CATAAGGGTCCTGTTTAACTGGCGGTGCGGTGTCCTTTTGACAAGTTACA
61	7007-7106	Cs- CTTCTGACATGTAATAGCTCTGGACTGCAAAAACCTATATGTGTCTTCTA<br>Rs- AAGGATAACTTGGCATAGCGATCCTCCTTGGGCGGCGGGGCAGCAGCACC
62	6722-6821	Cs- ACAAACAGTTCATTAAACCAACAAATACCATTATTATGGCCCTGCGCCCG<br>Rs- AGGCGGTACAAATAGTAAAATTAGTACTCCTAGTAGTATCCACCACAGTA
64[$]	6447-6543	Cs- GTTAGAAACAATTGATTATGCCAACAAATTCCATTCTTATGTCCCTGTGC<br>Rs- GTGTGCCTACACAAACAGAAAAGTTTGTACTTCTGGTAGTATCAACAACA
66	6617-6716	Cs- GTTGGTGCTTCTGGTAGTATCCACAACAGTAACAAATACCTGATTACCCC<br>Rs- TCACGGGCATCATATTTAGTTAATGTGCTTTTAGCTGCATTAATAGTCAT
67	6771-6870	Cs- CAGTCCTCTAATATATCTGGATTCATGGTGTGTATGTATTGCATAACATT<br>Rs- TATATGTGTCCTGTAAATTACCTGAAGGAGGTGGTGTAAGGCCAAATTGC
68	6644-6743	Cs- ATCATCCAAAATAGCAGGATTCATAGTATGTATATATGACATTACATCAG<br>Rs- TATGTATCTACAAGACTAGCAGATGGTGGAGGGGCAACACCAAAATTCCA
68b[%]	6485-6584	Cs- TAAAATTGGTACTGCGAGTGGTATCCACAACAGTAAGAAATAATTGATTA<br>Rs- ATTAGGATCATAAATATTTGGTACAGCTGATTCAGTAGTAGTAGACAAAG

69	6517-6616	Cs- GTTGGTACTGCGGGTAGTATCTACACAAGTAACAAACAATTGGTTGCCCC<br>Rs- GGTTTAAAAGTGGCAGATGCAGATTGTGCAGATACAGTACTAATAGTGAG
70	6557-6656	Cs- ATTAGTACTACGTGTAGTGTCCACCACAGTAATAAACAACTGGTTATGCC<br>Rs- GGGCTATATACAGCAGGTATGGCCGTTTCGGTGCAGGCAGACAATGTAAA
71	6819-6918	Cs- ATTTGTACTACGTGATGTGTCCACAACTGTTACAAAAAGCAGATTGCCCC<br>Rs- CTAGAGGCTTTATATGTAGACTCAACAGTTTTGGTAGCACAGATGGACAT
72	7043-7142	Cs- GGCAGCCCCCTTTTGACAGGTAATGGCACGAGACTGCAAAAACCTATAGG<br>Rs- TCCAAAAGGATAAGTTAGCATATGGATCTTCTTTAGGAGGAGGGGT
73	6714-6813	Cs- GTGATGTTACATATCTATATGTTTCCTCTAAAGTACCTGACGGTGGTGGG<br>Rs- TGGGTCCTCTGTTTCTTTAGGAGGTTGAGGACGTTGGCAACTAATAGCCT
74	6552-6651	Cs- GTTAGTACTACGTGTGGTATCCACAACTGTAACAAACAATTGATTACCCC<br>Rs- AGGAGGGGATTGTGTAGTAGGCGCACACACTGTCAT
81	6837-6936	Cs- ATTGGTGCTTCTGGTAGTATCCACCACTGTAACAAACATTTCATTAAACC<br>Rs- TTAGAGGCCTTGTATTCTGCAGCAGCAGATGTAGCTGTGCAAATAGTAAA
82	6799-6898	Cs- TTGCTGCATTTTTTACAAATCGATAGGCATCCTCCAAACTAGCGGAGG<br>Rs- GTCTTCTTTAGCCTGTGGAGGACTGTCCTTGTGACAGGATG
82_ IS39*	6699-6798	Cs- TTGCTGCATTTTTCACAAAGCGATAGGCATCCTCCAAACTAGCTGAAG<br>Rs- TTTAGCCTGTGGAGGGCTGTCCCGTTGACAAGAGG
83	6969-7068	Cs- GCACGGGACTGCAGATAGCGATAGGTATCATCAAGGCTGGTGGAGGGAG<br>Rs- CTTTTTAGGGGCAGGGGCGGAAGGACCCTTTTGGCAGGTAATA
84	6647-6746	Cs- ATTGGTGCTGCGGGTGGTATCTACCACCGTGACAAACAATTGATTAAACC<br>Rs- TTGGTAGGTTTATATTCTGATTCGGTGTTGGTAGCAGCACTAATAGTAAA
87	6760-6859	Cs- TGGCAGCACTAATAGTAAAATTGGTACTGCGAGTAGTATCAACAACCGTT<br>Rs- CCTTAAATATTCCTTAAACTTTGTGGGGTCATATTCAGTGGTTGTTTGAG
89	6723-6822	Cs- GGGTGGTATCCACCACAGTAACAAACAACTCATTAAACCAACAAATACCA<br>Rs- CCAGACTGGGAAGCAGCACAAATGGTAAGGTTGGTACTAC
90	6774-6873	Cs- ATTGGTGCTACGTGTAGTATCAACCACAGTTACAAAAAGCTGATTACCCC<br>Rs- TTGGAAGCCTTGTATGTGTCAGAGGGTGTTTGTGTGGCACAAATAGTCAT
91	6838-6937	Cs- GTTAGTGCTGCGAGTTGTATCCACAACAGTTACAAACACCTGGTTACCAA<br>Rs- TTGTCATATGTAGTAGGTAGCACAGACTCAGTGGATGCACACAAGGTTAA
114	7148-7247	Cs- GGCGCGGGACTGTAGATACCTGTAGGTGTCATCCAAACTT<br>Rs- GTAGGCTTGGCAGGAGCAGCCCCCTTCTGGCAGGAAAT
*HBB***	1912-2011	Cs- GGCTGGGCATAAAAGTCAGGGCAGAGCCATCTATTGCTTA<br>Rs- AATCTACTCCCAGGAGCAGGGAGGGCAGGAGCCAG

[&]Reference sequence: GeneBank accession No.: U31791.1

[$]Reference sequence: GeneBank accession No.: AJ812226 (HPV34)

[%]Reference sequence: GeneBank accession No.: FR751039.1

*Reference sequence: GeneBank accession No.: AF293961.1

**Reference sequence: GeneBank accession No.: GU324922.1

Table S4. Details on samples tested with No-PCR

Sample Number	Sample Source	Collection/ Fixaton	DNA extraction method
NS1	Cervical	SurePath	Automated: Chemagic extractor
NS2	Cervical	SurePath	Automated: Chemagic extractor
NS3	Cervical	**SurePath**	Automated: Chemagic extractor
NS4	Cervical	SurePath	Automated: Chemagic extractor
NS5	Cervical	SurePath	Automated: Chemagic extractor
NS6	Cervical	STM	Automated: Chemagic extractor
NS7	Cervical	STM	Automated: Chemagic extractor
NS8	Cervical	STM	Automated: Chemagic extractor
NS9	Cervical	STM	Automated: Chemagic extractor
NS10	Cervical	STM	Automated: MagnaPure
NS11	CervicoVaginal	Dry Swab	Manual: QiaAmp
NS12	CervicoVaginal	Dry Swab	Manual: QiaAmp
NS13	CervicoVaginal	Dry Swab	Manual: QiaAmp
NS14	CervicoVaginal	Dry Swab	Manual: QiaAmp
NS15	CervicoVaginal	Dry Swab	Manual: QiaAmp
NS16	Anal	Swab in STM	Automated: MagnaPure
NS17	Anal	Swab in STM	Automated: MagnaPure
NS18	Anal	Swab in STM	Automated: MagnaPure
NS19	Anal	Swab in STM	Automated: MagnaPure
NS20	Anal	Swab in STM	Automated: MagnaPure
NS21	Anal	Swab in STM	Automated: MagnaPure
NS22	Anal	Swab in STM	Automated: MagnaPure
NS23	Anal	Swab in STM	Automated: MagnaPure
NS24	Anal	Swab in STM	Automated: MagnaPure
NS25	Anal	Swab in STM	Automated: MagnaPure
NS26	Cervical	FFPE	Manual: Qiagen DNeasy
NS27	Cervical	FFPE	Manual: Qiagen DNeasy
NS28	Cervical	FFPE	Manual: Qiagen DNeasy
NS29	Cervical	FFPE	Manual: Qiagen DNeasy
NS30	Cervical	FFPE	Manual: Qiagen DNeasy
NS31	Cervical	FFPE	Manual: Qiagen DNeasy
NS32	Cervical	FFPE	Manual: Qiagen DNeasy
NS33	Cervical	FFPE	Manual: Qiagen DNeasy
NS34	Cervical	FFPE	Manual: Qiagen DNeasy
NS35	Cervical	FFPE	Manual: Qiagen DNeasy
NS36	Cervical	FFPE	Manual: Qiagen DNeasy
NS37	Cervical	FFPE	Manual: Qiagen DNeasy
NS38	Cervical	FFPE	Manual: Qiagen DNeasy
NS39	Cervical	FFPE	Manual: Qiagen DNeasy
NS40	Cervical	FFPE	Manual: Qiagen DNeasy
NS41	5 HPV plasmid (11, 16, 31, 45, 52) pool (10000 copies each)		
NS42	5 HPV plasmid (11, 16, 31, 45, 52) pool (1000 copies each)		
NS43	5 HPV plasmid (11, 16, 31, 45, 52) pool (100 copies each)		
NS44	5 HPV plasmid (11, 16, 31, 45, 52) pool (10 copies each)		
NS45	HPV16 positive SiHa cell line DNA (100ng)		
NS46	HPV16 positive SiHa cell line DNA (10ng)		
NS47	Placental DNA (100ng in 50ul TE)		
NS48	H2O control		

Table S5. Details of samples tested with PCR-15 and HPV TypeSeq

Sample number*	Sample Source	Collection/Fixation	DNA Extraction
PG63*	Anal	Self-collected swab in STM	Automated: Magna Pure
PG64*	Anal	Self-collected swab in STM	Automated: Magna Pure
PG65*	Anal	Self-collected swab in STM	Automated: Magna Pure
PG85*	Cervix	FFPE	Automated: Chemagic extractor
PG86*	Cervix	FFPE	Automated: Chemagic extractor
PG87*	Cervix	FFPE	Automated: Chemagic extractor
PG88*	Cervix	FFPE	Automated: Chemagic extractor
PG89*	Cervix	FFPE	Automated: Chemagic extractor
PG90*	Cervix	FFPE	Automated: Chemagic extractor
PG91*	Cervix	FFPE	Automated: Chemagic extractor
PG92*	Cervix	FFPE	Automated: Chemagic extractor
PG93*	Cervix	FFPE	Automated: Chemagic extractor
PG94*	Cervix	FFPE	Automated: Chemagic extractor
PG95*	Cervix	FFPE	Automated: Chemagic extractor
PG96*	Cervix	FFPE	Automated: Chemagic extractor
PG97*	Cervix	FFPE	Automated: Chemagic extractor
PG98*	Cervix	FFPE	Automated: Chemagic extractor
PG99*	Cervix	FFPE	Automated: Chemagic extractor
PG100	Cervix	FFPE	Automated: Chemagic extractor
PG101	Cervix	FFPE	Automated: Chemagic extractor
PG102	Cervix	FFPE	Automated: Chemagic extractor
PG103	Cervix	FFPE	Automated: Chemagic extractor
PG104	Cervix	FFPE	Automated: Chemagic extractor
PG105*	Cervicovaginal	Dry swab	Manual-Qiagen
PG106*	Cervicovaginal	Dry swab	Manual-Qiagen
PG107*	Cervicovaginal	Dry swab	Manual-Qiagen
PG108*	Cervicovaginal	Dry swab	Manual-Qiagen
PG109*	Cervicovaginal	Dry swab	Manual-Qiagen
PG110*	Cervicovaginal	Dry swab	Manual-Qiagen
PG111*	Cervicovaginal	Dry swab	Manual-Qiagen
PG112	Cervicovaginal	Dry swab	Manual-Qiagen
PG113	Cervicovaginal	Dry swab	Manual-Qiagen
PG114	Cervicovaginal	Dry swab	Manual-Qiagen
PG115	Cervicovaginal	Dry swab	Manual-Qiagen
PG116	Cervicovaginal	Dry swab	Manual-Qiagen
PG117	Cervicovaginal	Dry swab	Manual-Qiagen
PG118	Cervicovaginal	Dry swab	Manual-Qiagen
PG119	Cervicovaginal	Dry swab	Manual-Qiagen
PG120	Cervicovaginal	Dry swab	Manual-Qiagen
PG121	Cervicovaginal	Dry swab	Manual-Qiagen
PG122	Cervicovaginal	Dry swab	Manual-Qiagen
PG123	Cervicovaginal	Dry swab	Manual-Qiagen
PG124	Cervicovaginal	Dry swab	Manual-Qiagen
PG125*	Cervix	SurePath	Automated: Chemagic extractor
PG126*	Cervix	SurePath	Automated: Chemagic extractor
PG127*	Cervix	SurePath	Automated: Chemagic extractor
PG128*	Cervix	SurePath	Automated: Chemagic extractor
PG129*	Cervix	SurePath	Automated: Chemagic extractor
PG130*	Cervix	SurePath	Automated: Chemagic extractor
PG131*	Cervix	SurePath	Automated: Chemagic extractor
PG132	Cervix	SurePath	Automated: Chemagic extractor
PG133	Cervix	SurePath	Automated: Chemagic extractor
PG134	Cervix	SurePath	Automated: Chemagic extractor
PG135*	Anal	Self-collected swab in STM	Automated: Magna Pure
PG136*	Anal	Self-collected swab in STM	Automated: Magna Pure
PG137*	Anal	Self-collected swab in STM	Automated: Magna Pure
PG138b*	Anal	Self-collected swab in STM	Automated: Magna Pure
PG139*	Anal	Self-collected swab in STM	Automated: Magna Pure
PG140	Anal	Self-collected swab in STM	Automated: Magna Pure
PG141	Anal	Self-collected swab in STM	Automated: Magna Pure
PG142	Anal	Self-collected swab in STM	Automated: Magna Pure
PG143	Anal	Self-collected swab in STM	Automated: Magna Pure

*A subset of samples (n=44) marked with asterisk sign in this table were also tested by HPV TypeSeq assay

PG144b	Anal	Self-collected swab in STM	Automated: Magna Pure
PG145	Anal	Self-collected swab in STM	Automated: Magna Pure
PG146	Anal	Self-collected swab in STM	Automated: Magna Pure
PG147	Anal	Self-collected swab in STM	Automated: Magna Pure
PG148	Anal	Self-collected swab in STM	Automated: Magna Pure
PG149	Anal	Self-collected swab in STM	Automated: Magna Pure
PG150*	Pool of seven HPV plasmids (6,16,18,31,33,45, 52) each in 50 copies/reaction		
PG151*	Pool of seven HPV plasmids (6,16,18,31,33,45, 52) each in 25 copies/reaction		
PG152*	Pool of seven HPV plasmids (6,16,18,31,33,45, 52) each in 10 copies/reaction		
PG153*	SiHa DNA(10ng/reaction)		
PG154*	HeLa DNA (10 ng/reaction)		
PG155*	Placenta l DNA (100ng/reaction)		
PG156*	Water control		

Table S6: L1 consensus primer targeting 450 bp PCR product

PGMY Primer names	Primer sequence (5' → 3')
PG11-A	GCA CAG GGA CAT AAC AAT GG
PG11-B	GCG CAG GGC CAC AAT AAT GG
PG11-C	GCA CAG GGA CAT AAT AAT GG
PG11-D	GCC CAG GGC CAC AAC AAT GG
PG11-E	GCT CAG GGT TTA AAC AAT GG
PG9-F	CGT CCC AAA GGA AAC TGA TC
PG9-G	CGA CCT AAA GGA AAC TGA TC
PG9-H	CGT CCA AAA GGA AAC TGA TC
PG9-I	G CCA AGG GGA AAC TGA TC
PG9-J	CGT CCC AAA GGA TAC TGA TC
PG9-K	CGT CCA AGG GGA TAC TGA TC
PG9-L	CGA CCT AAA GGG AAT TGA TC
PG9-M	CGA CCT AGT GGA AAT TGA TC
PG9-N	CGA CCA AGG GGA TAT TGA TC
PG9-P	G CCC AAC GGA AAC TGA TC
PG9-Q	CGA CCC AAG GGA AAC TGG TC
PG9-R	CGT CCT AAA GGA AAC TGG TC
HMB01	GCG ACC CAA TGC AAA TTG GT
RSMY09-L	CGT CCT AAT GGG AAT TGG TC
Globin primer names	Primer sequence
GH20	GAA GAG CCA AGG ACA GGT AC
PC04	CAA CTT CAT CCA CGT TCA CC

E6 Results	L1 Results		Total
	+	-	
+	192	64	256
-	55	1945	2000
Total	247	2009	2256

Table S7. Type-specific concordance between results for E6 and L1 CodeSets for direct NanoString testing (No-PCR assay)*

*Agreement, 94.73% (2137/2256); k=0.734, considered substantial agreement; McNemar P = 0.463; Total type-sample combinations, 47 types x 48 samples = 2256.

Table S8. Type-specific concordance of HPV CodeSets in comparison to LA or TypeSeq (Z-test results) under different testing and sample conditions

Level of comparison	PCR-45 vs LA (1)	PCR-15 vs LA (2)	PCR-15 vs TypeSeq (3)[&]	p-value (Z-test)[$]
**Overall agreement**				
All 37 LA types	0.962	0.957	0.949	1 vs 2= 1.00<br>2 vs 3 = 1.00
HR types	0.971	0.962	0.956	1 vs 2 = 1.00<br>2 vs 3 = 1.00
HPV16/18	0.999	0.993	0.966	1 vs 2 = 1.00<br>2 vs 3 = 0.714
**Kappa values**				
All 37 LA types	0.862<br>(95% CI 0.828-0.898)	0.796<br>(95% CI 0.761-0.832)	0.77<br>(95% CI 0.736-0.888)	1 vs 2= 0.534<br>2 vs 3 = 1.00
HR types	0,91<br>(95% CI 0.867-0.953)	0.848<br>(95% CI 0.802-0.894)	0.862<br>(95% CI 0.811-0.912)	1 vs 2 =0.515<br>2 vs 3 = 1.00
HPV16/18	1.00<br>(95% CI 1.00-1.00)	0.979<br>(95% CI 0.939-1.00)	0.913<br>(95% CI 0.817-1.00)	1 vs 2 =0.939<br>2 vs 3 = 0.225

[&]All 47 types common between the PCR-15 and TypeSeq are included in this comparison

[$]All Z-test p-values are >0.05 indicating no significant difference between proportions compared indicating reproducible performance of CodeSets for HPV detection compared to two other methods (LA or TypeSeq), under different assay conditions.
